# Role of Hsp100/Clp Protease Complexes in Controlling the Regulation of Motility in *Bacillus subtilis*

**DOI:** 10.3389/fmicb.2016.00315

**Published:** 2016-03-16

**Authors:** Noël Molière, Jörn Hoßmann, Heinrich Schäfer, Kürşad Turgay

**Affiliations:** ^1^Naturwissenschaftliche Fakultät, Institut für Mikrobiologie, Leibniz Universität HannoverHannover, Germany; ^2^Institut für Biologie-Mikrobiologie, Freie Universität BerlinBerlin, Germany

**Keywords:** AAA+ proteins, regulatory proteolysis, ClpC, ClpX, ClpP, motility, *Bacillus subtilis*

## Abstract

The Hsp100/Clp protease complexes of *Bacillus subtilis* ClpXP and ClpCP are involved in the control of many interconnected developmental and stress response regulatory networks, including competence, redox stress response, and motility. Here we analyzed the role of regulatory proteolysis by ClpXP and ClpCP in motility development. We have demonstrated that ClpXP acts on the regulation of motility by controlling the levels of the oxidative and heat stress regulator Spx. We obtained evidence that upon oxidative stress Spx not only induces the thiol stress response, but also transiently represses the transcription of flagellar genes. Furthermore, we observed that in addition to the known impact of ClpCP via the ComK/FlgM-dependent pathway, ClpCP also affects flagellar gene expression via modulating the activity and levels of the global regulator DegU-P. This adds another layer to the intricate involvement of Clp mediated regulatory proteolysis in different gene expression programs, which may allow to integrate and coordinate different signals for a better-adjusted response to the changing environment of *B. subtilis* cells.

## Introduction

Hsp100/Clp proteases are compartmentalized protein degradation machines, which consist of a peptidase component (i.e., ClpP) and an AAA+ ATPase (i.e., ClpC or ClpX). The peptidase subunits are arranged in a barrel-like double heptamer with the catalytic residues on the inside surface of the structure. Folded proteins are excluded from the catalytic sites because they are too large to fit through the opening of the pore and are thus protected from proteolysis. The AAA+ ATPases form a hexameric ring with a narrow pore, which associates with one or both sides of the peptidase barrel. Specific substrate proteins can be recognized by the N-terminal ATPase domain, often facilitated by adaptor proteins, and are unfolded and threaded through the pore by the AAA+ ATPase motor into the peptidase chamber, where they are degraded (Kirstein et al., 2009; Sauer and Baker, 2011).

Hsp100/Clp proteases participate in general and regulatory proteolysis in the bacterial cell. For example, the ClpCP complex in *Bacillus subtilis* acts in protein quality control by degradation of unfolded, misfolded or aggregated proteins, which accumulate under stress conditions such as heat shock (Krüger et al., 2000; Schlothauer et al., 2003). Interestingly, the same protein complex plays an important part in developmental processes by controlled degradation of transcription factors like the competence master regulator ComK (Turgay et al., 1998), the class III heat shock repressor CtsR (Derré et al., 1999; Krüger et al., 2001; Kirstein et al., 2007) and the anti-anti sigma factor SpoIIAB involved in sporulation (Pan et al., 2001). ClpCP may also play a role in the processing of SlrR, a newly identified regulator of biofilm formation (Chai et al., 2010). ClpE is homologous to ClpC, with the exception of the N-terminal domain, which is homologous to the N-terminal domain of ClpX (Kirstein et al., 2009). ClpE appears to be important under severe heat shock conditions (Miethke et al., 2006).

An important regulatory substrate of the third *B. subtilis* Hsp100/Clp ClpXP protease is the thiol and oxidative stress transcription factor Spx (Nakano M. M. et al., 2002; Nakano S. et al., 2002). Under non-stress conditions, Spx is very efficiently turned over by ClpXP aided by the adaptor protein YpbH, resulting in a low steady state concentration of the protein. When cells encounter oxidative or heat stress, *spx* transcription is up-regulated (Helmann et al., 2001; Leelakriangsak et al., 2007). More importantly, the Spx protein is stabilized either by oxidative inactivation (Garg et al., 2009) or heat-mediated sequestration (Engman and von Wachenfeldt, 2015) of the adaptor protein YjbH, leading to rapid accumulation of the active regulator (Zuber, 2009; Runde et al., 2014).

Spx is a transcriptional regulator, which forms a complex with the C-terminal domain of the RNA polymerase alpha subunit (alpha-CTD; Nakano et al., 2003b; Newberry et al., 2005). By enhancing RNA polymerase interaction with certain promoters, Spx can serve as an activator i.e., of genes encoding enzymes required to cope with thiol oxidative stress (Nakano et al., 2003a; Reyes and Zuber, 2008). Interestingly, Spx can act as a transcriptional repressor on another group of genes (Nakano et al., 2003a,b). According to the interference model, genes, which require an activator that binds to the RNA polymerase alpha-CTD, are repressed because Spx competes with binding of the activators to the alpha-CTD (Nakano et al., 2003b; Zhang et al., 2006).

Another interesting process, in which Clp proteases appear to be involved, is the regulation of swimming motility in *B. subtilis* (Mukherjee and Kearns, 2014). Already during their initial characterization, *clpP, clpC*, and *clpX* mutant strains were reported to be non-motile (Rashid et al., 1996; Liu and Zuber, 1998; Msadek et al., 1998). However, the mechanisms, by which Clp proteases affect swimming motility, are currently only partially understood. Swimming or swarming bacterial cells are propelled by flagella, rotating filamentous helical structures, which are powered by an intra-membrane revolving motor. Gene regulation of flagellar assembly is a hierarchical process as described for *Escherichia coli* (Chevance and Hughes, 2008) and *B. subtilis* (Mukherjee and Kearns, 2014). No obvious flagellar master regulator such as FlhDC of *E. coli* has been identified in the *B. subtilis* genome, instead, the early flagellar genes (class II genes) are located in a single large *fla/che* operon (Márquez-Magaña and Chamberlin, 1994). This operon is transcribed by the σ^A^ housekeeping sigma factor (Kearns and Losick, 2005) and is modulated by a number of transcription factors including DegU (Amati et al., 2004; Tsukahara and Ogura, 2008), CodY (Bergara et al., 2003), and SwrA (Kearns and Losick, 2005; Calvio et al., 2008). The *sigD* gene encoding the alternative sigma factor σ^D^ is positioned close to the 3′-end of the *fla/che*-operon (Márquez-Magaña and Chamberlin, 1994; Cozy and Kearns, 2010). The class III or late flagellar genes include *hag*, which encodes flagellin, the major structural subunit of the flagellum. They are organized in separate transcriptional units controlled by σ^D^-dependent promoters (Márquez et al., 1990). σ^D^ is inhibited by its anti-sigma factor FlgM, which is an important morphogenetic checkpoint synchronizing gene expression with the assembly of the flagella (Mirel et al., 1994; Fredrick and Helmann, 1996; Bertero et al., 1999; Chevance and Hughes, 2008; Mukherjee and Kearns, 2014; Calvo and Kearns, 2015).

How could regulatory proteolysis by Hsp/100Clp proteins act on motility development? Liu et al. could demonstrate that high ComK concentrations in *clpC* or *mecA* mutant cells result in a transcriptional read-through from *comFA* into *flgM*. This leads to over-production of FlgM, which inhibits σ^D^ and represses *hag* transcription and thus motility development (Liu and Zuber, 1998). However, another study has proposed a second *comK*-independent effect of a *clpC* mutant on motility (Rashid et al., 1996). In addition, the proteolysis substrates responsible for the effect of *clpX* on swimming motility are unknown to date.

Here, we analyzed the influence of regulatory proteolysis on swimming motility in detail and identified two transcriptional regulators, which inhibit swimming motility and are affected by Clp proteases. We found that ClpCP, in addition to its control of the ComK mediated induction of FlgM expression (Liu and Zuber, 1998), also affects DegU~P mediated inhibition of motility. Most interestingly, we observed that Spx, a proteolysis substrate of ClpXP, negatively regulates motility genes by an unknown, probably indirect mechanism. Thereby heat or oxidative stress signals sensed by ClpXP/Spx can result in a halt of motility in *B. subtilis* cells.

## Materials and methods

### General methods

*B. subtilis* cells were cultured in Luria–Bertani (LB) medium (5 g/l yeast extract, 10 g/l tryptone–peptone, 5 g/l NaCl) at 37°C if not otherwise indicated. Overnight cultures were inoculated from freshly streaked colonies and grown in LB medium in the presence of appropriate antibiotics (10 μg/ml chloramphenicol, 1 μg/ml erythromycin + 25 μg/ml lincomycin, 10 μg/ml kanamycin, 10 μg/ml tetracycline, or 100 μg/ml spectinomycin). Standard DNA manipulation was carried out as described previously (Sambrook et al., 2001). Protein concentrations were determined using the Bradford method (Bradford, 1976).

### Cloning

Cloning was performed in *E. coli* XL-1 blue cells (Stratagene). Phusion High Fidelity DNA Polymerase (New England Biolabs) was used for PCR amplifications. Chromosomal DNA was used as a template. Restriction enzymes and T4 DNA Ligase were obtained from Fermentas. Primer sequences are listed in Table S3, plasmids are listed in Table S2.

Plasmids pQE60-hag and pQE60-spx were constructed by amplification of the *hag* or *spx* genes using primers hagpQE60-for and hagpQE60-rev or spxpQE60-for and spxpQE60-rev, respectively and cloning into plasmid pQE60 (Qiagen) using the *Nco*I and *BamH*I restriction sites. Plasmids pX-hag1 and pX-hag4 were obtained by PCR amplification using primers hag1-for and hag1-rev or hag4-for and hag4-rev, respectively, *BamH*I digestion and ligation into *BamH*I-digested pX plasmid. For plasmid pflgB152, the *flgB152* promoter fragment was amplified using primers flgB152-for and flgB152-rev and cloned into plasmid pDG268 using the *EcoR*I and *BamH*I restriction sites. All plasmids were sequenced.

### Transformation/strain construction

All strains used in this study are described in Table S1. Transformation with chromosomal DNA or plasmid DNA was performed by a standard method (Anagnostopoulos and Spizizen, 1961). Strains BNM421 and BNM426 were constructed by transformation of strain BNM126 (Δ*hag*) with plasmids pX-hag1 or pX-hag4, respectively. BNM421 expresses the *hag* gene from the xylose-inducible P_xyl_ promoter. In BNM426, a *hag* fragment, comprising 92 bases upstream and 32 bases downstream of the open reading frame, is under the control of P_xyl_. Strain BNM426 complemented the swimming motility defect of the Δ*hag* mutant in the presence of xylose.

BNM109 was constructed using the technique of long-flanking homology PCR as described previously (Wach, 1996) with the primers listed in Table S3. Strains BNM126 and BNM149 were constructed as described using plasmids pMADhag and pMADcomK as described previously (Arnaud et al., 2004; Blair et al., 2008). Δ*clpP*, Δ*clpC*, and Δ*clpX* mutants were obtained by transformation of the recipient strains with chromosomal DNA from strains BNM103, BNM105, and BNM106, respectively. Strains BNM350 and BNM351 were constructed by transformation of strain BNM111 (Δ*spx::kan*) with plasmids pMMN521 or pSN56 (Nakano et al., 2003a), respectively. Strain BNM810 was acquired by transformation of the wild type strain with plasmid pSN56. The strains BNM1266, BNM1268, and BNM 1270 were constructed by transforming the *B. subtilis*168 *swrA*^+^ *degQ*^+^ (Gift of Nicola Stanley-Wall) with chromosomal DNA prepared from BNM103, 105, or 109 (Table S1).

To obtain strain BNM866, chromosomal DNA from strain ABH282, featuring a second copy of *amyE* at the *ywrK* gene locus (Camp and Losick, 2009), was first transformed into the wild type strain 168, resulting in strain BNM860. BNM860 was then transformed with plasmid pSN56 (Nakano et al., 2003a) selecting for spectinomycin resistance and chloramphenicol sensitivity, which indicates integration of the *spx*^DD^ construct into the *ywrK::amyE* locus (BNM866). This strain produced Spx protein after isopropyl-β-D-thiogalactopyranoside (IPTG) induction, as verified by Western blot analysis (data not shown). Finally, to yield strains BNM878 and BNM1001, strain BNM866 was transformed with chromosomal DNA from strains BNM301 (*amyE::PflgB*-*lacZ cat*) or BNM328 (*amyE::Phag*-*lacZ cat*) selecting for chloramphenicol and spectinomycin resistance, indicating integration of the *PflgB*-*lacZ* or *Phag*-*lacZ* constructs at the original *amyE* locus, while the spx^DD^ construct remains at the *ywrK:amyE* locus. We confirmed by Western blot analysis that the resulting strains produce Spx protein in response to IPTG induction (data not shown).

### Pulse chase labeling and immunoprecipitation

Cells were grown in Belitsky minimal medium [50 mM Tris-(hydroxymethyl)-amino methane (Tris) -HCl pH 7, 5, 15 mM (NH_4_)_2_ SO_4_, 8 mM MgSO_4_, 27 mM KCl, 7 mM sodium citrate, 0.6 mM KH_2_PO_4_, 2 mM CaCl_2_, 160 μg/ml L-tryptophan, 10 μM MnSO_4_, 1 μM FeSO_4_, 4.5 mM potassium glutamate, 0, 2% glucose] to OD_600_ 0.7 at 37°C. 3.5 ml bacteria were removed and pulse labeled with 30 μCi L-^35^S-methionine for 10 min at 37°C. Subsequently, cold L-methionine (0.3 M) was added in 30-fold excess and samples were taken after the indicated incubation times and mixed with trichloroacetic acid (TCA) to a final concentration of 10% w/v. TCA-precipitated samples were incubated on ice for 10 min and centrifuged for 15 min at 17,000 g and 4°C. The pellets were washed twice in 1 ml acetone, air-dried and resuspended in 20 μl lysis buffer (50 mM Tris-HCl pH 7.5, 5 mM EDTA, 1 mM PMSF, 4 mg/ml lysozyme). The samples were boiled for 3 min at 95°C. Two hundred and seventy microliters KI buffer (50 mM Tris-HCl pH 8, 150 mM NaCl, 0.5% Triton X-100, 1 mM PMSF) was added and the samples were incubated on ice for 15 min. Precipitate was separated by centrifugation for 15 min at 17,000 g at 4°C. Hundred microliters of the supernatant were mixed with 2 μl polyclonal anti-Hag antiserum and incubated over night at 4°C for immunoprecipitation. The next day, 8 μl Protein A Magnabeads (Thermo Scientific) were added to the solution and mixed. The magnetic beads were washed twice in 200 μl KI buffer. Subsequently, the magnetic beads were resuspended in SDS sample buffer, boiled for 3 min at 95°C and applied to 12.5% SDS PAGE gels. Electrophoresis was performed at 25 mA per gel for 1 h and the gels were vacuum dried on Whatman paper for 2 h at 85°C. The dried gels were placed on a phosphoimager screen for 24 h and screens were scanned using a Fla 2000 phosphoimager (Fujifilm, Japan).

### Motility assay

Overnight cultures grown in LB medium at 37°C were diluted to OD_600_ 2.0 in fresh LB medium and 3 μl were applied to tryptone agar plates containing 0.3% w/v agar (bacteriology grade, Carl Roth), 10 g/l tryptone/peptone and 5 g/l NaCl. The plates were incubated at 37°C for 8 h. The growth behavior of all examined strains in liquid culture with the tryptone salt medium (utilized for the swimming plates) was comparable to growth in LB (data not shown).

### Protoplast preparation

Thirty milliliters of a growing *B. subtilis* culture were harvested by centrifugation and the pellets were washed twice in 1 ml STM (50 mM Tris-HCl pH 8, 50 mM NaCl, 5 mM MgCl_2_, 25% w/v sucrose). Subsequently, the pellets were re-suspended in 200 μl STM buffer + 0.3 mg/ml lysozyme and incubated for 30 min at 37°C to obtain protoplasts. The protoplasts were washed twice in 1 ml STM and then lysed by resuspension in 200 μl TM buffer without sucrose (50 mM Tris-HCl pH 8, 50 mM NaCl, 5 mM MgCl_2_) containing 10 μg/ml DNase I and 10 μg/ml RNase A and incubated on ice for 30 min. The lysate was centrifuged for 20 min at 17,000 g and 4°C and the supernatant was transferred to a fresh tube. Total protein concentration was determined by the Bradford assay.

### Whole cell preparation

One milliliter of a growing *B. subtilis* culture was harvested by centrifugation and the pellet was washed twice in 1 ml STM (50 mM Tris-HCl pH 8.0, 50 mM NaCl, 5 mM MgCl_2_, 25% w/v sucrose) + 0.3 mg/ml lysozyme and incubated for 5 min at 37°C. 6x SDS sample buffer containing SDS and DTT was added and the samples were boiled at 95°C for 5 min. The samples were centrifuged for 5 min at 17,000 g prior to SDS–PAGE separation.

### Western blot analysis

Polyclonal antibodies against Hag and Spx were produced in rabbits by inoculation with purified Hag-His_6_ or Spx-His_6_ (Pineda Antibody Services, Berlin, Germany). Rabbit-anti-Spx antibodies for initial experiments were kindly provided by Peter Zuber (University of Oregon). SigD antibodies from rabbit were kindly provided by John Helmann (Cornell University), rabbit-anti-CodY antibodies from Linc Sonenshein (Tufts University), and sheep-DegU antibodies from Nicola Stanley–Wall (University of Dundee).

Lysates from protoplasts were adjusted to equal concentration and 2.5–10 μg per lane total protein were loaded onto 12.5 or 15% SDS-gels and separated by electrophoresis. Gels were blotted onto PVDF membranes in 20 mM Tris-HCl pH 8.3, 150 mM glycine, 20% v/v methanol using a semi-dry blotting chamber. Blot membranes were blocked in TBS-M (50 mM Tris-HCl pH 8.0, 150 mM NaCl, 5% w/v skim milk) and incubated with antisera diluted in TBS-M. Antisera were used at dilutions of 1:40,000 (anti-Hag), 1:5000 (anti-DegU, anti-SigD, anti-Spx), or 1:10,000 (anti-CodY). The blots were washed in TBS buffer (50 mM Tris-HCl pH 8.0, 150 mM NaCl) and incubated with secondary anti-rabbit (GE Healthcare) or anti-sheep (Sigma Aldrich) antibodies conjugated to alkaline phosphatase diluted 1:10,000 in TBS-M. The blot membranes were then equilibrated in AP buffer (100 mM Tris-HCl pH 9.5, 100 mM NaCl, 5 mM MgCl_2_) and developed using ECF Western Blotting Reagent (GE Healthcare). The fluorescence signals were scanned using a Fla 2000 phosphoimager (Fujifilm, Japan).

### β-galactosidase assay

One to five milliliters samples of a growing *B. subtilis* carrying a *lacZ* fusion were collected and harvested by centrifugation for 5 min at 17,000 g and frozen at −20°C. For the β-galactosidase measurement, the cell pellets were thawed on ice and resuspended in 500 μl Z buffer (100 mM NaPO_4_ pH 7.0, 1 mM MgSO_4_, 100 mM β-mercaptoethanol). Ten microliters toluene was added and the samples were thoroughly mixed and incubated on ice for 30 min. For the assay, the samples were diluted 4-fold and transferred into a flat-bottom 96 well plate (final volume 200 μl). The reaction was started by addition of 50 μl ONPG (4 mg/ml in Z buffer) using an 8-channel multi-pipette (Eppendorf). Absorbance at 420 nm was measured every 60 s for 15 min at room temperature using a microplate reader (Tecan Instruments). The β-galactosidase activity (in Miller Units) was calculated from the linear slope of the absorbance at 420 nm over time correcting for the sample path length in the microplates. For comparison of β-galactosidase activities of strains exhibiting lag phases in growth (i.e., Δ*clpP* and Δ*clpX* mutants), the time axis was normalized to T0, the point of deviation from exponential growth.

### *Spx*^DD^ induction

Strains BNM351 (Δ*spx::kan amyE::P*_*Hy*_-*spx*^DD^) and BNM350 (Δ*spx::kan amyE::P*_*Hy*_-*spx*) were grown in LB medium at 37°C to OD_600_ 0.3. Subsequently, the culture was split and expression of *spx*^DD^ was induced by addition of 1 mM IPTG to one half of the culture. Samples were withdrawn before addition of ITPG (0 min), 30 and 60 min thereafter and total RNA or total protein were prepared as described above for Northern or Western blot analysis.

### Thiol oxidative stress experiments

A growing culture was divided in early exponential phase and 1 mM N,N,N′,N′-Tetramethyl-azodicarboxamide (diamide) was added to half of the culture to induce thiol oxidative stress. Samples were removed before addition of diamide at the indicated time points and β-galactosidase activity was determined. Cell lysates of the same samples were analyzed by SDS–PAGE and Western blot against Spx.

### Northern blot analysis

All buffers used for RNA work were treated with 0.1% diethyl pyrocarbonate (DEPC) and autoclaved (121°C, 20 min). RNA was prepared from 30 ml *B. subtilis* cultures using the FastRNA Pro Blue kit (MP Biochemicals). Lysis was performed by shaking in a Retsch mill for 10 min at 1800 rpm. The RNA was digested with RNase free DNase I (Roche Applied Sciences) to remove contaminating DNA and subsequently purified by phenol–chloroform extraction and ethanol precipitation. The RNA concentration was determined by absorbance measurement at 260 nm using a NanoDrop spectrophotometer (Peqlab).

RNA samples were diluted in DEPC treated H_2_O, mixed 1:1 with 2x RNA sample buffer (60 mM MOPS–NaOH pH 7.0, 0.02% w/v Bromphenol blue, 75% v/v formamide, 3.33% w/v formaldehyde, 3% w/v Ficoll 70) and heated to 65°C for 10 min. Ten microliters RNA Molecular Weight Marker III (Roche Applied Sciences) was applied to the gel as a size standard. The RNA was separated by electrophoresis on 1.2% w/v agarose gels in 40 mM MOPS–NaOH pH 7.0, 5 mM sodium acetate, 1 mM EDTA, 0.1% w/v diethyl pyrocarbonate (DEPC) and 37% v/v formaldehyde for 2 h and 45 min at 80 V. The gel was rinsed with 20x SSC (300 mM tri-sodium citrate pH 7.0, 3 M NaCl, 0.1% DEPC) and vacuum blotted onto a positively charged nylon membrane (Roche Applied Sciences) in 10x SSC (150 mM tri-sodium citrate pH 7.0, 1.5 M NaCl, 0.1% DEPC) for 1.5 h at 5 mm Hg pressure. UV crosslinking was performed for 10 min at 328 nm. Subsequently, the blot was stained in methylene blue solution (0.02% w/v methylene blue, 300 mM sodium acetate pH 5.5, 0.1% DEPC) for 5 min to visualize ribosomal RNAs as a control for equal sample application and blotting. The membrane was destained in Bleaching buffer (0.2x SSC 1% w/v SDS, 0.1% DEPC) and equilibrated in 2x SSC (30 mM tri-sodium citrate pH 7.0, 0.3 M NaCl, 0.1% DEPC).

Digoxigenin (DIG) labeled DNA probes were prepared by PCR using PCR DIG labeling mix containing DIG-dUTP (Roche Applied Sciences). PCR was performed with Phusion High Fidelity DNA polymerase (New England Biolabs) using primers hag-probe-for and hag1-rev (see Table S3). A first round of PCR was performed with chromosomal DNA as a template in the absence of DIG labeling mix. The product of this reaction was used as a template for a second round of PCR in the presence of DIG labeling mix. The PCR products were purified by gel extraction using the ZymoClean™ Gel DNA recovery kit (Hiss Diagnostics) and eluted in 20 μl DEPC treated H_2_O. The probes were denatured for 5 min at 95°C and cooled rapidly on ice.

The nylon membrane was transferred to a hybridization glass tube and incubated with 20 ml DIG Easy Hybridization solution (Roche Applied Sciences) for 1 h at 47°C with rotation in a hybridization oven. Twenty microliters DIG-labeled probe (100 ng/μl) was diluted in 20 ml DIG Easy Hybridization solution and the membrane applied to the blot over night at 47°C. The blot was washed twice with 20 ml wash buffer 1 (0.1% w/v SDS, 30 mM tri-sodium citrate pH 7.0, 0.3 M NaCl, 0.1% v/v DEPC) for 5 min at 47°C and twice with wash buffer 2 (0.1% w/v SDS, 1.5 mM tri-sodium citrate pH 7.0, 15 mM NaCl, 0.1% v/v DEPC) for 30 min at 47°C.

The blot membrane was blocked in Blocking buffer [100 mM maleic acid pH 7.5, 150 mM NaCl, 1% w/v Blocking reagent (Roche Applied Sciences)] for 30 min at room temperature. Anti-digoxigenin antibodies conjugated to alkaline phosphatase (Roche Applied Sciences) were diluted 1:5000 in Blocking buffer and applied to the blot for 1.5 h at room temperature. Subsequently, the blot was washed twice for 15 min in Detection buffer 1 (100 mM maleic acid pH 7.5, 150 mM NaCl) and equilibrated in Detection buffer 2 (100 mM Tris-HCl pH 9.5, 100 mM NaCl, 5 mM MgCl_2_) for 2 min. CDP Star solution (Roche Applied Sciences) was applied to the blot and the signal was detected using X-ray films.

### Electrophoretic mobility shift assays

DNA probes were produced by PCR amplification from chromosomal DNA using the primer sets flgB (−209 to −6)-for/flgB (−209 to −6)-rev flgB (−106 to 98)-for/flgB (−106 to 98)-rev and flgB (−1 to 203)-for/flgB (−1 to 203)-rev (see Table S3) and purified by gel extraction.

Fifty nanograms of the DNA probes were mixed with purified Spx-His_6_ at 1.25, 2.5, 5 μM protein concentration in TSM buffer (10 mM Tris-HCl pH 7.5, 1 mM EDTA, 5% v/v glycerol, 1 mM MgCl_2_, 10 mM NaCl) in the presence of 1 μg poly-d(I–C; Roche Applied Sciences) and incubated for 20 min at room temperature. Subsequently, the samples were applied to 5% w/v polyacrylamide gels and electrophoresis was performed for 2 h at 80 V in TSM buffer. The gels were stained with ethidium bromide and bands were visualized by UV illumination.

## Results

### Clp proteases affect regulation of swimming motility

**Figure 1:**
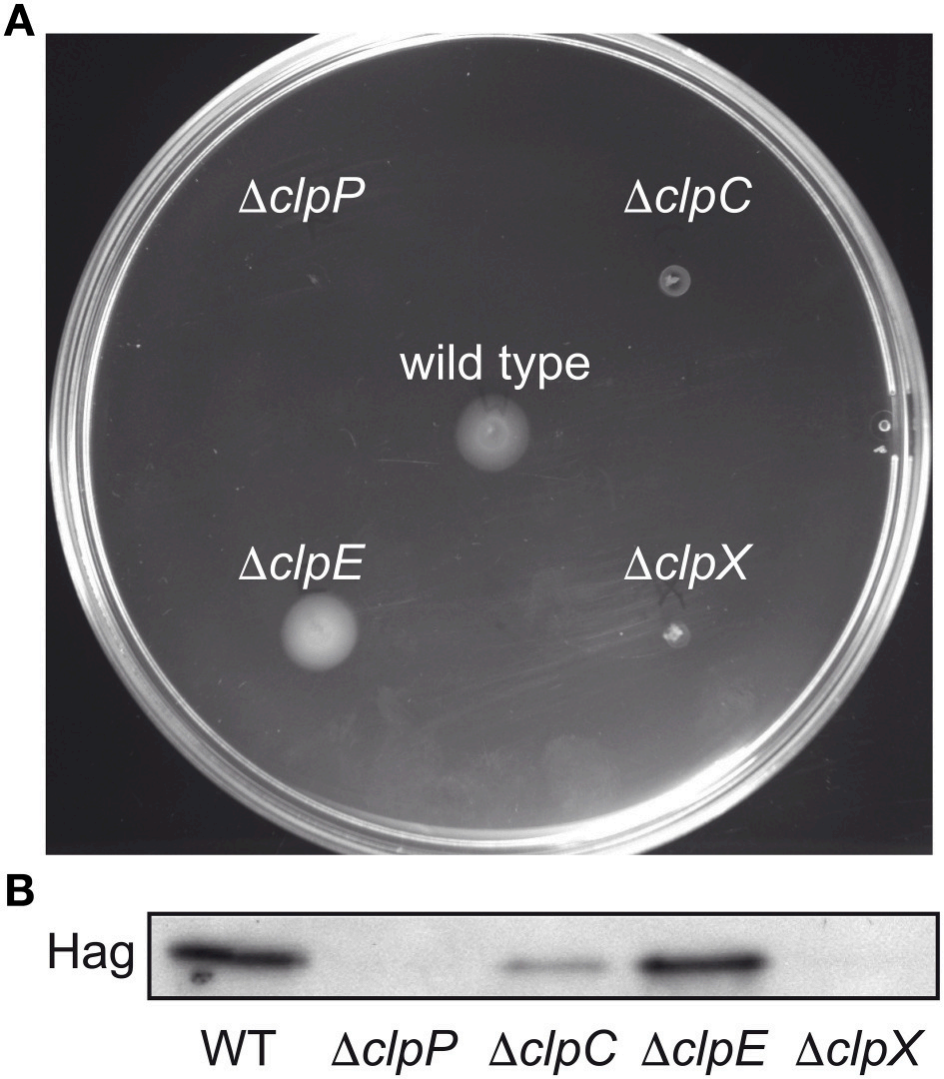
**Clp proteases affect swimming motility and Hag protein levels in ***B. subtilis*****. **(A)** Motility assay of *B. subtilis* wild type and Δ*clpP::spec* (BNM103), Δ*clpC::tet* (BNM105), Δ*clpE::spec* (BNM106), and Δ*clpX::kan* (BNM107) mutants on 0.3% w/v agar plates. **(B)** Cells of the indicated strains (as in **A**) were grown to OD_600_ 1.0 at 37°C. Cell lysates were analyzed by SDS–PAGE and Western blotting using anti-Hag antibodies.

We examined the swimming motility of wild type and *clp* mutant *B. subtilis* cells and confirmed that *clpP* and *clpC* mutant strains exhibit a defect in swimming motility (Rashid et al., 1996; Liu and Zuber, 1998; Msadek et al., 1998). In addition we observed that a *clpX* mutant is non-motile, whereas a *clpE* mutant displayed similar motility to the wild type (Figure 1A).

We observed that the overall Hag protein level, the major Flagellin protein in *B. subtilis*, appears to be relatively stable, judging by *in vivo* by pulse chase experiments following immuno-precipitation in wild type *B. subtilis* cells (Figure S1). When we examined Hag levels in Clp mutant strains we observed very low Hag levels in *clpC* (Rashid et al., 1996) and no detectable Hag protein in *clpX* and *clpP* mutant strains (Figure 1B).

To investigate whether the observed effect of the mutants on Hag protein level is reflected in the mRNA levels of flagellar transcripts, we performed Northern blot experiments using a probe against the *hag* transcript. We observed that hag mRNA levels were strongly reduced in *clpP, clpC*, and *clpX* mutant strains (Figure S2). We noted an additional band of ~500 bases hybridizing with our probe in the *clpC* and *hag* mutants (Figure S2). This might be explained by an upregulation of the shorter *B. subtilis yvzB* transcript, which encodes a smaller homolog of Hag, in these mutants.

We conclude from these data that the absence of Clp proteases resulted in a swimming motility defect and strongly diminished Hag protein and transcript levels (Figure 1 and Figure S2), suggesting that ClpCP and ClpXP indirectly affect Hag protein levels, i.e., by regulatory proteolysis of transcriptional regulators controlling the synthesis of Hag.

### Clp proteases regulate transcription from the *flgB* promoter

We first aimed to determine, which process in flagellar biogenesis is affected by the *clp* mutations. To this end, we performed reporter gene assays using transcriptional fusions of flagellar promoters to *lacZ* to elucidate whether transcription initiation from these promoters is altered in *clp* mutant strains. One construct (*PflgB*-*lacZ*) contains the upstream sequence of the *fla/che* operon from residues −479 to +47 relative to the transcriptional start site of the σ^A^-dependent *flgB* promoter (P_A_) and is indicative of flagellar class II gene expression (Kearns and Losick, 2005). The minor σ^D^-dependent promoter, which does not influence flagellar gene expression (Kearns and Losick, 2005), is also present in this sequence (P_D3_). To monitor the σ^D^-dependent class III genes we used a transcriptional *lacZ* fusion to the *hag* promoter (*Phag*-*lacZ*) fusion (Kearns and Losick, 2005). All *lacZ* fusions were integrated into the ectopic *amyE* locus. We introduced *clp* mutations into these strains and determined β-galactosidase activities of samples along the growth curve.

**Figure 2:**
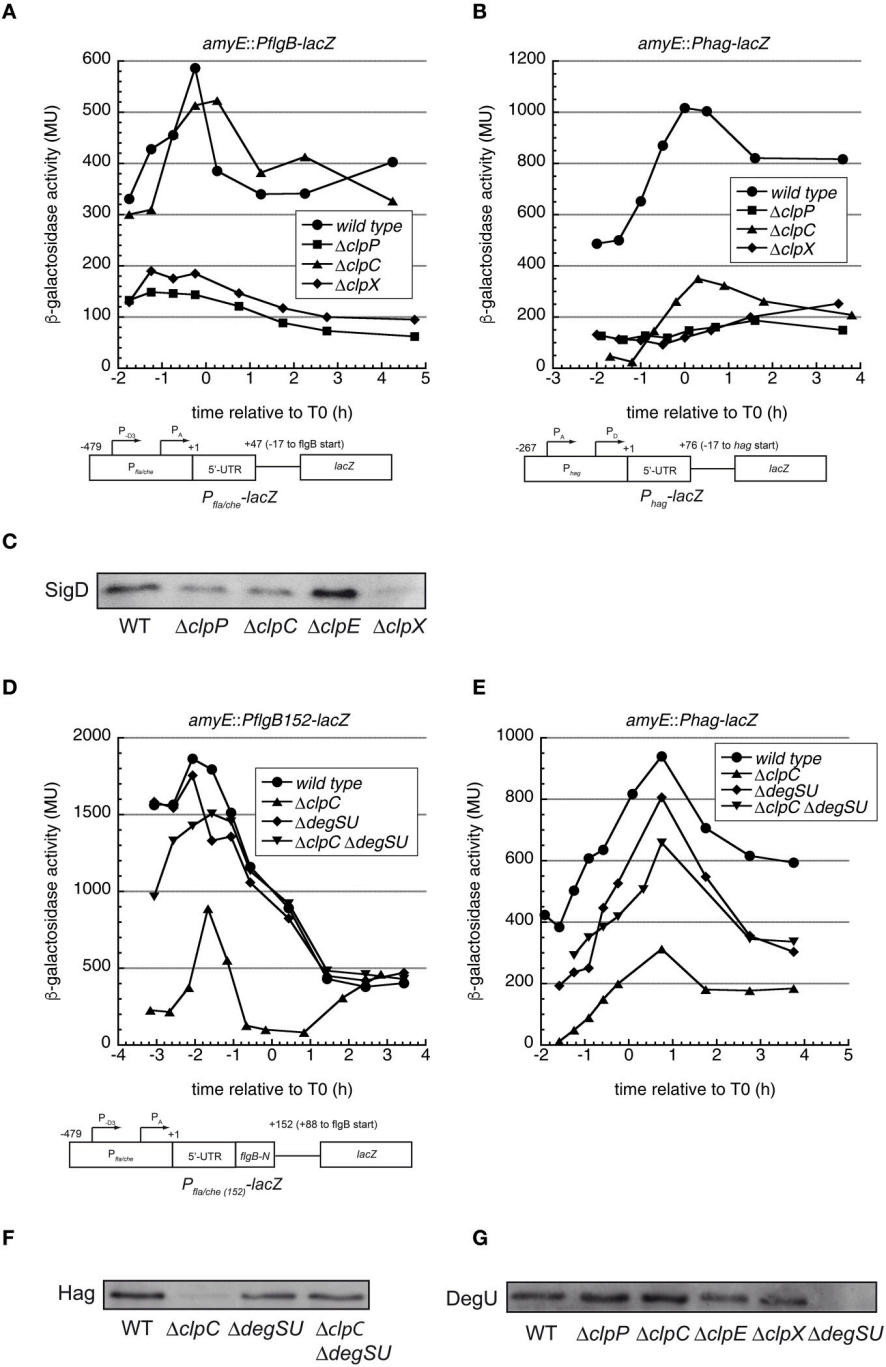
**Motility genes are down-regulated in ***B. subtilis clp*** mutant cells and the role of ***clpC*** and ***degSU*****. **(A)** β-galactosidase assays of the indicated strains carrying a *PflgB*-*lacZ* fusion. Circles: wild type 168 (BNM301), squares: Δ*clpP::spec* (BNM302), triangles: Δ*clpC::tet* (BNM303), diamonds: Δ*clpX::kan* (BNM305). Representative data from at least two independent experiments are shown. A schematic drawing of the promoter-lacZ fusion is depicted at the bottom. **(B)** Same as **(A)** for the *hag* promoter *lacZ* fusion. Circles: wild type 168 (BNM328), squares: Δ*clpP::spec* (BNM329), triangles: Δ*clpC::tet* (BNM330), diamonds: Δ*clpX::kan* (BNM332). **(C)** Cells of strains wild type, BNM103 (Δ*clpP*), BNM105 (Δ*clpC*), BNM107 (Δ*clpX*) were grown to OD_600_ 1.0 at 37°C. Cell lysates were analyzed by SDS–PAGE and Western blotting using anti-SigD **(D)** β-galactosidase assays of the indicated strains carrying a *PflgB152*-*lacZ* fusion Circles: wild type 168 (BNM346), triangles: Δ*clpC::tet* (BNM347), diamonds: Δ*degSU::spec* (BNM348), inverted triangles: Δ*clpC::tet* Δ*degSU::spec* (BNM349). Representative data from at least two experiments are shown. A schematic drawing of the promoter-lacZ fusion is depicted at the bottom. **(E)** Same as **(D)** for the *hag* promoter *lacZ* fusion. Circles: wild type (BNM328), triangles: Δ*clpC* (BNM330), diamonds: Δ*degSU* (BNM333) and inverted triangles: Δ*clpC* Δ*degSU* (BNM338). **(F)** Cells of the wild type and strains BNM105 (Δ*clpC*), BNM138 (Δ*degSU*), and BNM140 (Δ*clpC* Δ*degSU*) were grown to OD_600_ 1.0 at 37°C. Cell lysates were analyzed by SDS–PAGE and Western blotting using anti-Hag antibodies. **(G)** Cells were grown at 37°C to T0 (time of deviation from exponential growth) and cell lysates of the wild type and strains BNM103 (Δ*clpP*), BNM105 (Δ*clpC*), BNM106 (Δ*clpE*), BNM107 (Δ*clpX*), and BNM138 (Δ*degSU*) were analyzed by SDS–PAGE followed by Western blotting using anti-DegU antibodies.

In the wild type, both *PflgB*-*lacZ* and *Phag*-*lacZ* expression displayed the typical pattern of flagellar genes with a peak in post exponential phase (Mirel and Chamberlin, 1989; Figures 2A,B). Notably, in the *clpP* and *clpX* mutants, but not the *clpC* mutant, *PflgB*-*lacZ* activity was strongly reduced throughout growth (Figure 2A). The *Phag*-*lacZ* fusion was strongly down-regulated in the *clpP, clpC*, and *clpX* mutants (Figure 2B).

These results indicate that ClpXP affects transcription from the *flgB* promoter, whereas the lack of ClpC might affect *hag* promoter activity. However, as an additional control, we performed Western blots to determine the protein levels of the flagellar sigma factor σ^D^, which is encoded in the *fla/che* operon and directly activates transcription from the *hag* promoter. As expected, *clpP* and *clpX* mutants exhibited lower σ^D^ protein levels, but the same was true for the *clpC* mutant (Figure 2C), even though *clpC* had no apparent effect on the activity of the tested *PflgB*-*lacZ* fusion (Figure 2A), suggesting that either *clpC* acts on σ^D^ post-transcriptionally or that additional elements near the *fla/che* promoter might be required for the observed down-regulation.

However, it was previously observed that the *flgB* promoter features two DegU binding sites, one located upstream of the promoter (BR1) and one downstream in the *flgB* coding region (BR2; Tsukahara and Ogura, 2008). The second BR2 element is not encoded in the *flgB* promoter LacZ fusion we used so far. Therefore, we constructed a longer *lacZ* fusion that included 17 additional bases upstream of the *flgB* start codon along with 88 bases of the *flgB* coding sequence (*PflgB152*-*lacZ*, see Materials and Methods and Figure 2D) including this additional DegU binding site (Tsukahara and Ogura, 2008). In the wild type background, this *lacZ* fusion displayed a similar expression pattern as the *PflgB*-*lacZ* construct, but peak expression was about three fold higher (Figure 2D). In addition, the *clpC* mutation had a strong negative effect on *lacZ* expression from this construct (Figure 2D). This suggests a possible role of DegU in the observed inhibition of swimming motility in the *clpC* mutant strain. In summary, our results suggest that transcription from the *flgB* promoter is strongly down-regulated in the *clpP, clpC*, and *clpX* mutants (Figures 2A,D), which results in lower protein levels of the flagellar sigma factor σ^D^ (Figure 2C). The lowered level of σ^D^ causes reduced transcription from the *hag* promoter (Figure 2B), lower Hag protein levels (Figure 1B) and reduced swimming motility (Figure 1A).

To further examine if the reduced Hag protein levels in *clp* mutants are solely a consequence of altered *hag* transcription, we uncoupled Hag production from σ^D^ regulation by placing a copy of *hag* downstream of the xylose-inducible P_xyl_ promoter at the ectopic *amyE* locus of a *hag* deletion mutant (Figure S3, see Section Materials and Methods). Interestingly, Hag levels were completely restored to wild type levels in the *clpC* background, implying that *clpC* acts on motility genes upstream of the *hag* promoter. In contrast, Hag levels were only partially restored in the *clpP* and *clpX* mutants, suggesting an additional effect of *clpP* and *clpX* on *hag* transcript or Hag protein levels downstream of transcription initiation from the *hag* promoter (Figure S3).

### ClpC influences swimming motility through ComK and DegU

Controlled proteolytic degradation of a regulatory protein appears to be an important mechanism, by which Clp proteases can influence gene expression as demonstrated e.g., for the control of competence development (Kirstein et al., 2009; Battesti and Gottesman, 2013). Liu and Zuber previously described a pathway, by which ClpCP regulates swimming motility through ComK, which positively influences the transcription of FlgM. Briefly, ComK activates competence genes, among them *comFA*, which is located directly upstream of *flgM* on the chromosome. In the absence of *clpC*, more FlgM is produced by transcriptional readthrough. FlgM inhibits σ^D^ activity, leading to decreased *hag* expression and reduced motility (Liu and Zuber, 1998).

To confirm that the reduced swimming motility of the *clpC* mutant is due to raised ComK levels, we tested the motility and Hag protein levels of the *clpC comK* double mutant strain. As shown in Figure S4, the *comK* mutation partially suppressed the swimming motility and Hag production defect of the *clpC* mutant. These results indicate that part of the motility defect of a *clpC* mutant is due to higher levels of ComK and supports the read-through transcription of *flgM* as suggested by Liu and Zuber (Liu and Zuber, 1998). However, FlgM is unlikely to play a part in the down-regulation of *flgB* promoter activity because FlgM specifically inhibits σ^D^ (Caramori et al., 1996), whereas *PflgB* transcription is independent of σ^D^ (Kearns and Losick, 2005).

Therefore, we examined other known repressors of the *fla/che* operon. One candidate is DegU, which can act as a repressor of the *fla/che* operon in its phosphorylated form (Amati et al., 2004). In addition, the DNA element between positions +48 and +152 relative to the transcription start site of the *flgB* promoter, which is required for the *clpC* mediated down-regulation of *fla*/*che* transcription (Figure 2D), contains a DegU~P binding site (Tsukahara and Ogura, 2008) and DegU~P has been described as a possible ClpCP substrate (Ogura and Tsukahara, 2010).

We tested whether DegU levels can be elevated in a *clpC* mutant. To this end we performed DegU Western blots in wild type and *clp* mutant strains at different time points during growth. Notably, only between T_0_ and T_2_, at a time when cells are motile and expressing flagellar genes, we detected mildly increased levels of DegU in the *clpC* and to a lesser extent in the *clpP* mutants compared to the wild type (Figure 2G and Figure S5).

**Figure 3:**
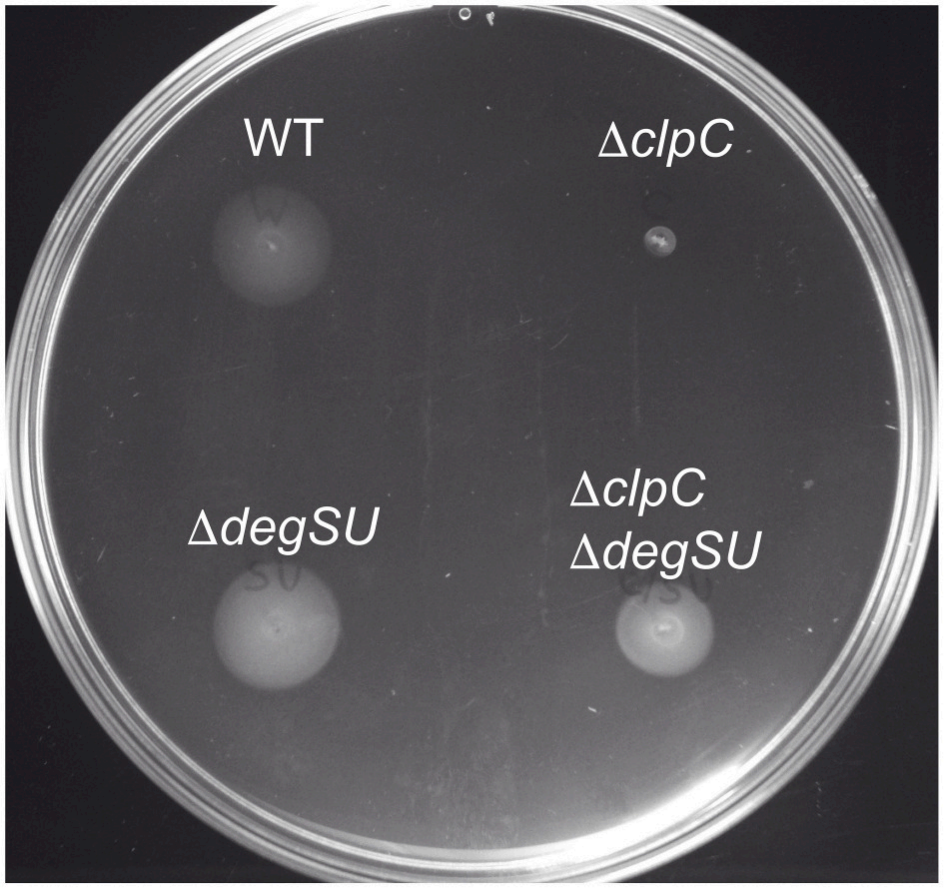
**A ***degSU*** mutation suppresses the swimming motility defect of a ***B. subtilis clpC*** mutant strain**. Motility assay of the wild type and strains BNM105 (Δ*clpC*), BNM138 (Δ*degSU*), and BNM140 (Δ*clpC* Δ*degSU*).

In order to test whether *degU* is responsible for the motility defect of the *clpC* mutant, we constructed a *clpC degSU* double mutant and tested swimming motility, Hag protein levels and motility gene expression of this strain. In this mutant, *degU* is deleted along with the *degS* gene, which encodes its cognate sensor kinase DegS. Indeed, the *degSU* mutation suppressed the swimming defect of the *clpC* mutant (Figure 3) and restored Hag production almost to wild type levels (Figure 2F), whereas mutation of *degSU* alone did not influence swimming and Hag concentration. Furthermore, β-galactosidase activity of the *flgB*-*lacZ* and to a little lesser extent of the *hag*-*lacZ* promoter reporter fusions was restored in the *clpC degSU* double mutant compared to the *clpC* mutant, but was significantly more similar to the wild type in the *degSU* mutant (Figures 2D,E). This suppression was specific to *clpC*, as the *degSU* mutation did not suppress down-regulation of the *hag*-*lacZ* promoter fusion and swimming motility in the *clpX* mutant (Figure S6). These results suggest that ClpC negatively influences DegU repressor activity.

We observed a growth dependent mildly raised level of DegU in a *clpC* and to a lesser extent in a *clpP* mutant strain (Figure S5), which is consistent with the hypothesis that under specific conditions ClpC could inhibit or ClpCP could also degrade DegU-P (Ogura and Tsukahara, 2010) and that, in the absence of *clpC*, active DegU-P can accumulate and thereby represses transcription from the *flgB* promoter (Figure 2D).

### Influence of the repressor cody on motility

The repressor CodY, which can sense GTP and branched chain amino acids, has been reported to bind to the *flgB* and *hag* promoters (Bergara et al., 2003; Ababneh and Herman, 2015). However, it was also observed that a *codY* deletion did not influence motility and *fla/che* expression (Amati et al., 2004) and a recent study, investigating the genome wide CodY binding sites did not detect CodY binding sites for controlling the *flgB* promoter (Belitsky and Sonenshein, 2013).

We could only detect small differences in motility of a *codY* mutant strain compared to wild type cells (Figure S7B) and we could not detect differences in the levels of cellular CodY protein in *clpC, clpX, clpE*, or *clpP* strains (Figure S7A). These results suggest that under our experimental conditions and in our strain background neither ClpCP nor ClpXP strongly influence motility via CodY.

### ClpXP regulate swimming motility through Spx

According to the data presented above, motility genes are strongly down-regulated in the *clpX* and *clpP* mutants. Interestingly, these mutants are phenotypically distinct from the *clpC* mutant: for example, the shorter *PflgB*-*lacZ* fusion was down-regulated in *clpX* and *clpP* mutants, but not in the *clpC* mutant. Furthermore, our data indicate that *clpX* and *clpP* act on swimming motility independently of *degU* (Figure S6). Therefore, we assumed that distinct substrates of ClpCP and ClpXP regulate motility.

Both *clpP* and *clpX* mutants have a slow growth phenotype, which leads to frequent acquisition of second site suppressor mutants. We isolated such suppressor mutants, which could be easily identified by larger colony size on plates and loss of the characteristic lag phase during growth in liquid medium (Figure S8A). Interestingly, we noticed that this strain was only slightly less motile than the wild type (Figure S8B) and produced wild type levels of Hag protein (Figure S8C). One well-characterized suppressor mutation of *clpX* and *clpP* mutants is a loss of function mutation in the *spx* gene, which relieves the detrimental effect of raised levels of the ClpXP substrate Spx (Nakano et al., 2001). We analyzed Spx levels by Western blot using polyclonal Spx antibodies and detected only very low levels of Spx in the wild type strain and no Spx in the *clpP* suppressor mutant, whereas Spx accumulated to high levels in a freshly transformed *clpP* mutant (Figure S8C). This strongly suggested that our isolated suppressor mutant of *clpP* is phenotypically similar to a *spx* mutant.

**Figure 4:**
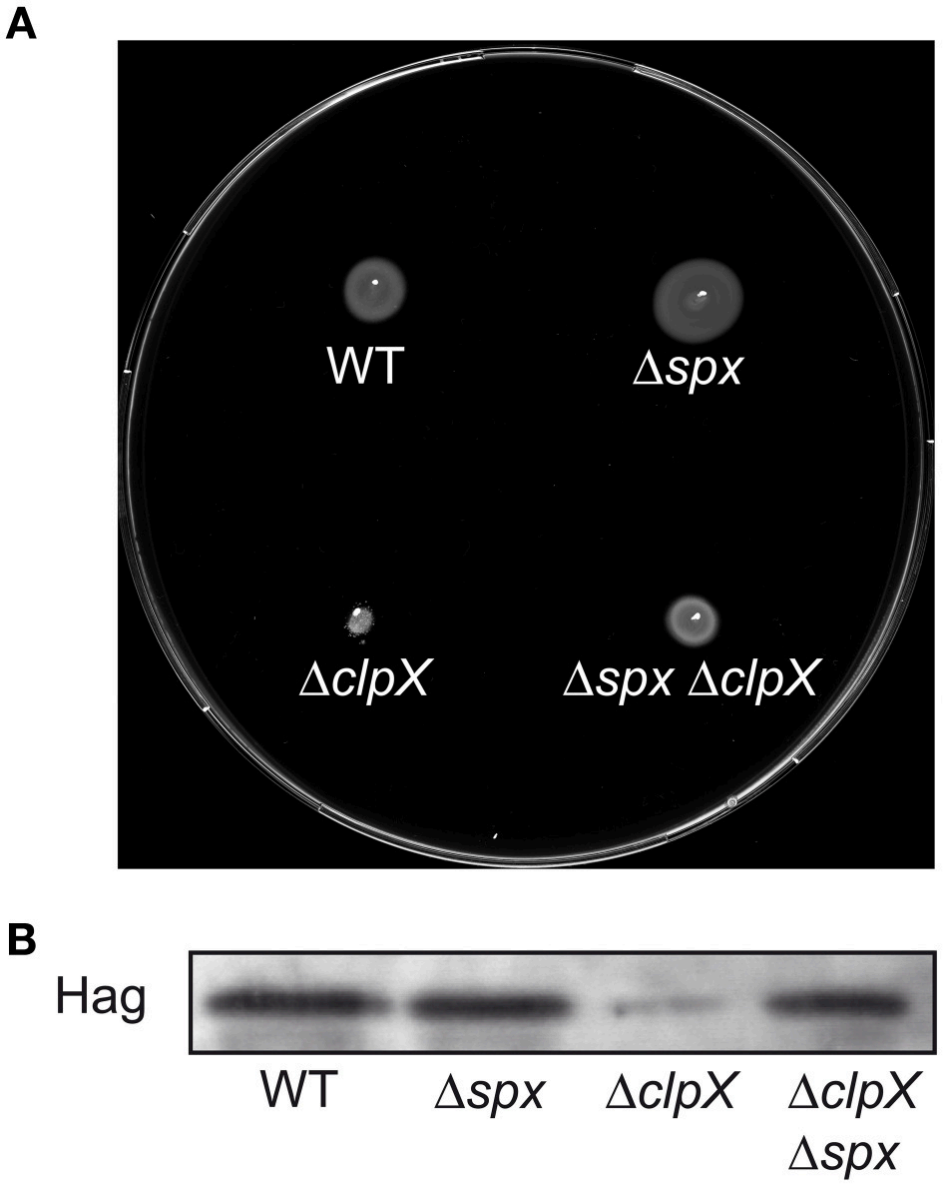
*****spx*** suppresses the ***swimming*** motility defect of a ***B. subtilis clpX*** mutant strain**. **(A)** Motility assay of the wild type and strains BNM107 (Δ*clpX*), BNM111 (Δ*spx*), and BNM112 (Δ*clpX* Δ*spx*). **(B)** Cells of the indicated strains (as in **A**) were grown to OD_600_ 1.0 at 37°C. Cell lysates were analyzed by SDS–PAGE and Western blotting using anti-Hag antibodies.

**Figure 5:**
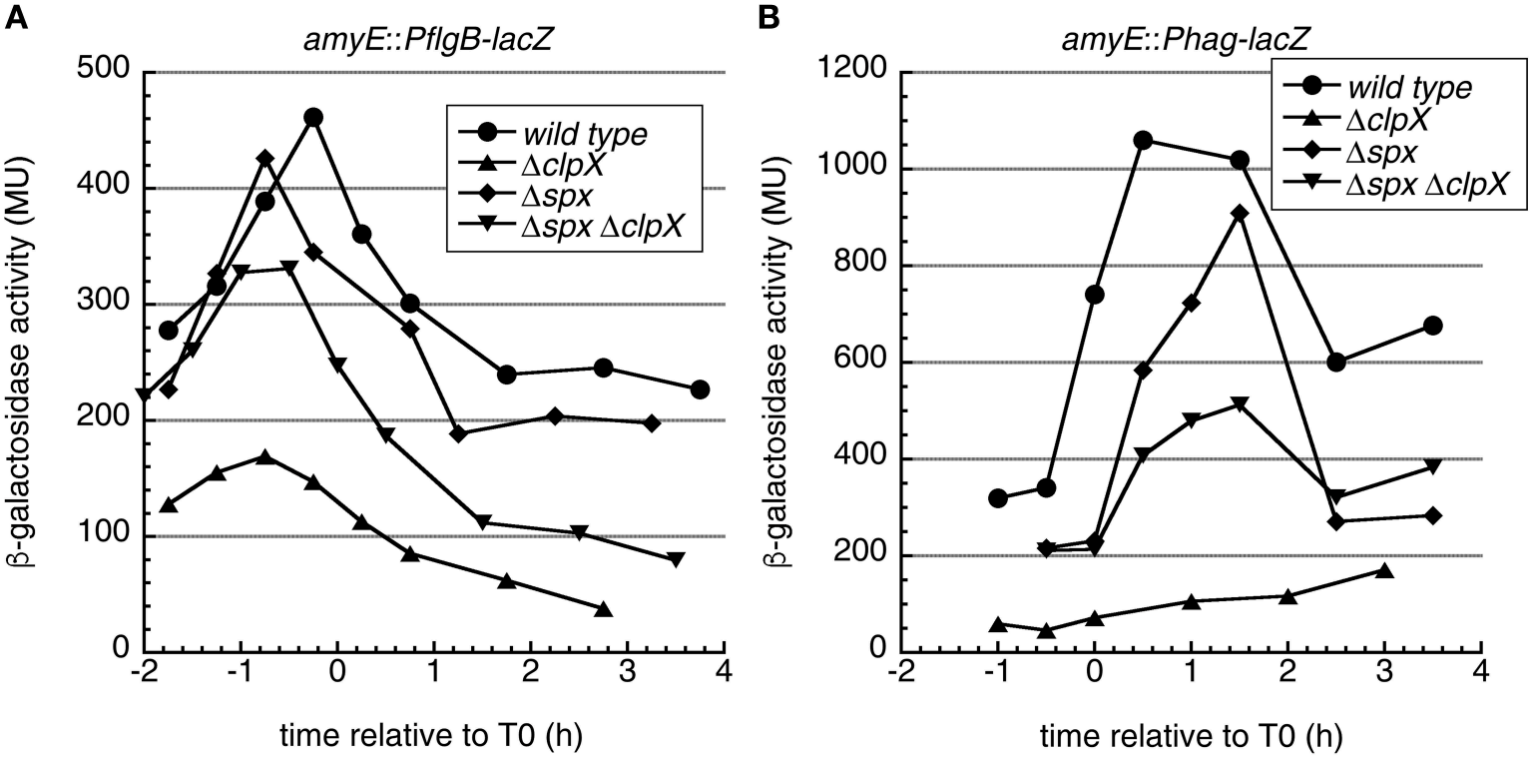
*****clpX*** affects motility gene expression via ***spx*****. **(A)** β-galactosidase assays of the indicated strains carrying a *PflgB*-*lacZ* fusion. Circles: wild type 168, triangles: Δ*clpX::kan* (BNM305), diamonds: Δ*spx::kan* (BNM307), inverted triangles: Δ*spx::kan* Δ*clpX::spec* (BNM308). Representative data from 2 to 3 experiments are shown. **(B)** Same as **(A)** for the *hag* promoter *lacZ* fusion. Circles: wild type 168, triangles: Δ*clpX::kan* (BNM332), diamonds: Δ*spx::kan* (BNM334), inverted triangles: Δ*spx::kan* Δ*clpX::spec* (BNM335).

We therefore tested swimming motility and Hag levels in a clean *clpX spx* double deletion mutant. Interestingly, the *spx* mutation resulted in increased motility on swim plates, whereas cellular Hag levels were similar to the wild type strain in this mutant (Figures 4A,B). Furthermore, the *spx* mutant suppressed the swimming motility defect of the *clpX* mutant and restored Hag production to wild type levels (Figures 4A,B). In addition, the activity of the *PflgB*-*lacZ* and *Phag*-*lacZ* fusions was partially restored in the *clpX spx* double mutant (Figures 5A,B), suggesting that the *flgB* promoter is to a large extent regulated by *clpX* via *spx*. The *spx* single mutant was significantly more similar to the wild type in these reporter gene assays (Figures 5A,B). Interestingly, we observed that the Hag levels were also restored in *clpX spx* mutant in a strain with xylose-controlled *hag* expression (Figure S3D), implying that the observed additional posttranscriptional effect of *clpX* on *hag* is *spx*-dependent.

### Spx negatively regulates motility genes

**Figure 6:**
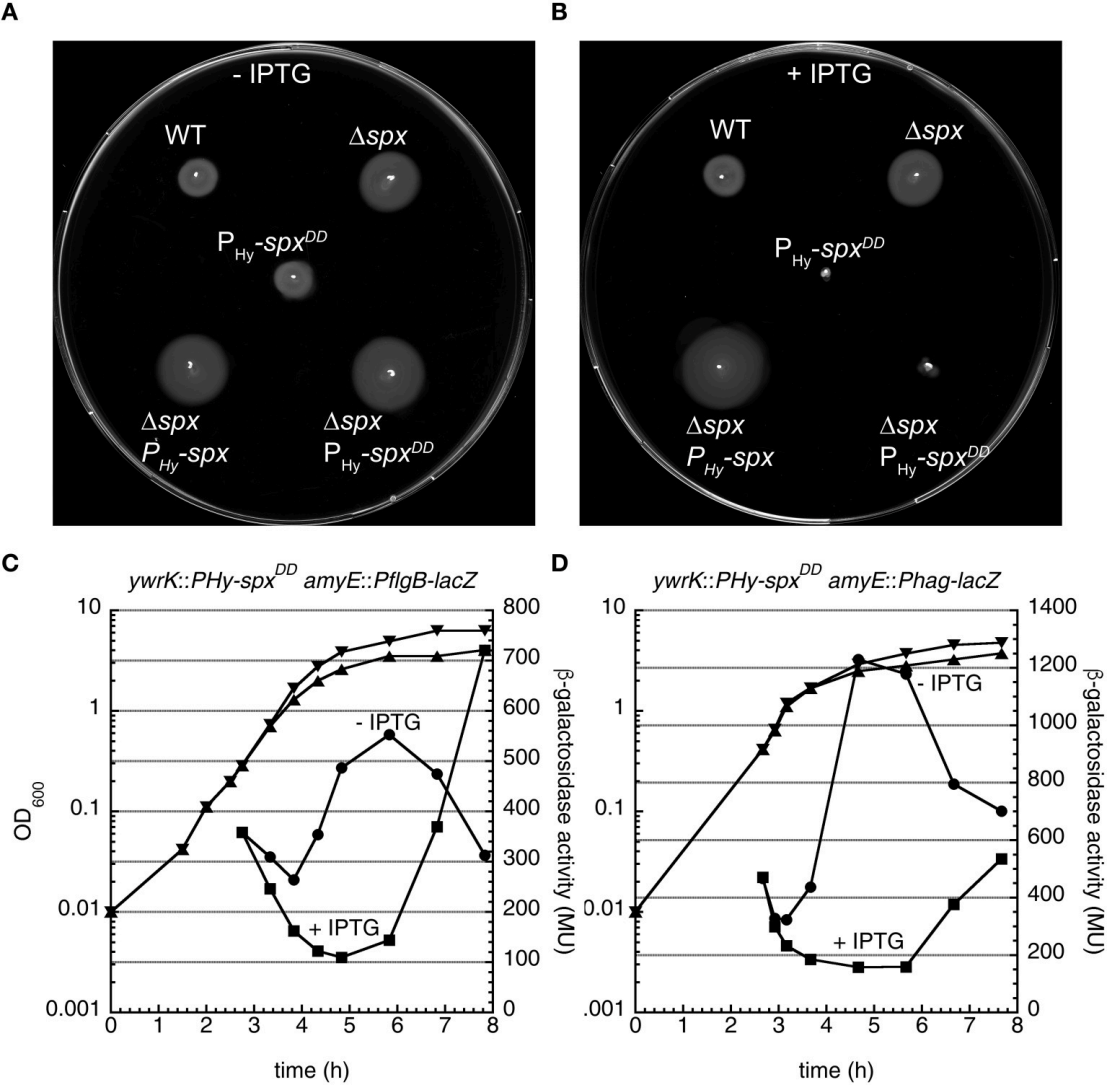
**Spx acts as a negative regulator of motility genes. (A)** Motility assay of the indicated strains [wild type: WT, BNM111: Δ*spx*, BNM810: *P*_Hy_-*spx*^DD^, BNM350 (Δ*spx P*_Hy_-*spx*), BNM351 (Δ*spx P*_Hy_-*spx*^DD^)]. Representative data from at least two experiments are shown. **(B)** Same as **(A)** using a swim agar plate containing 0.1 mM IPTG to induce *spx*^DD^. **(C)** A *B. subtilis* strain carrying both a transcriptional *flgB* promoter *lacZ* fusion at the *ywrK* locus and an IPTG-inducible copy of *spx*^DD^ at the *amyE* locus (BNM878) was grown in LB medium at 37°C and induced with 0.1 mM IPTG in early exponential phase (OD_600_ 0.2–0.3). β-galactosidase activity was measured at the indicated time points. Circles: control without IPTG, squares: induced with 0.1 mM IPTG, inverted triangles: OD_600_, no IPTG; triangles: OD_600_, induced with 0.1 mM IPTG. **(D)** Same as **(C)** using a strain with a *hag* promoter *lacZ* fusion at the *ywrK* locus combined with an IPTG-inducible copy of *spx*^DD^ at the *amyE* locus (BNM1001).

These results indicate that the reduced swimming motility of the *clpX* mutant might be caused by the presence of Spx, which negatively regulates the *flgB* promoter. To test whether Spx is able to inhibit motility also in a *clpX*^+^ background, we utilized a strain, in which a stabilized Spx variant (Spx^DD^) that can no longer be degraded by ClpXP, is encoded at the *amyE* locus under the control of an IPTG-inducible promoter (Nakano et al., 2003a). Notably, this strain was no more motile in the presence of IPTG, while an additional induction of wild type Spx had no effect on motility (Figures 6A,B), indicating that a raised level of Spx negatively regulates swimming motility.

In order to elucidate whether transcription from the *flgB* and *hag* promoters is regulated by *spx*^DD^ induction, we inserted the *PflgB*-*lacZ* or *Phag*-*lacZ* reporter fusions at an additional ectopic locus (*ywrK*) of a strain carrying an IPTG-inducible copy of *spx*^DD^ at the *amyE* locus (see Materials and Methods). We grew these strains to early exponential phase, induced *spx*^DD^ by addition of IPTG and determined β-galactosidase activity of these strains (Figures 6C,D).

The activity of both promoters was strongly repressed compared to the un-induced control for a period of ~3 h after induction and subsequently increased (Figures 6C,D). As an additional control, we analyzed the *hag* mRNA levels by Northern blot analysis and Hag protein levels by Western blot analysis of a strain carrying an IPTG-inducible copy of *spx*^DD^ at the *amyE* locus (Figure S9). The *hag* transcript level decreased below the detection limit of our Northern blot experiment after 30 min of *spx*^DD^ induction Figure S9A). Hag protein levels also decreased after *spx*^DD^ induction, however this effect was not as pronounced as for *hag* mRNA (Figure S9B), which is possibly due to the observed stability of Hag (Figure S1).

YjbH is an adapter protein, which specifically recognizes Spx and targets it for degradation by ClpXP. As an additional test to analyze the effect of increased Spx levels on motility, we assayed an *yjbH* deletion mutant for swimming motility and Hag levels. Similar to the *clpX* mutant, the *yjbH* mutant strain was unable to swim and displayed strongly decreased Hag levels. In contrast, an *spx yjbH* double mutant was highly motile and displayed wild type level of Hag protein (Figure S10).

Taken together, these data suggest that Spx acts as a negative regulator of swimming motility.

### Motility genes are down-regulated in response to thiol oxidative stress

Spx is present at very low concentrations in growing, non-stressed cells due to regulatory proteolysis by ClpXP and repression of the *spx* gene. In response to oxidative stress, *spx* is transcriptionally de-repressed (Leelakriangsak et al., 2007) and Spx is stabilized (Zhang and Zuber, 2007; Garg et al., 2009).

Since our results suggest that Spx acts as a negative regulator of motility, it is conceivable that motility is repressed under conditions, when Spx accumulates in the cell, such as during thiol oxidative stress. To test this we subjected the *PflgB*-*lacZ* and *Phag*-*lacZ* reporter strains to oxidative stress by addition of 1 mM diamide, a strong inducer of Spx activity and collected samples for determination of β-galactosidase activity.

**Figure 7:**
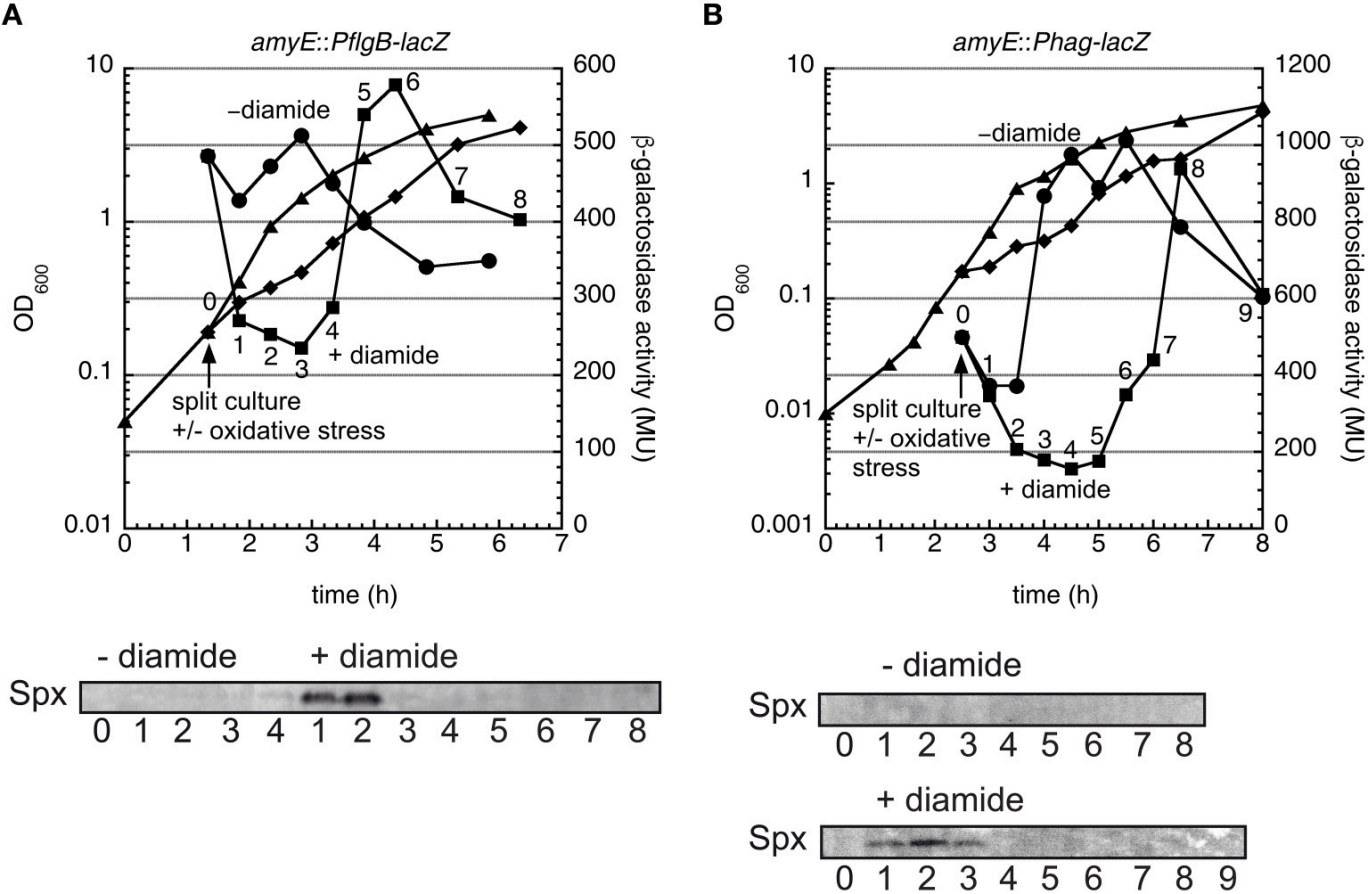
**Thiol oxidative stress results in transient down-regulation of motility genes in ***B. subtilis***. (A)** A growing culture of strain BNM301, carrying a transcriptional *PflgB*-*lacZ* fusion, was divided in early exponential phase and 1 mM diamide was added to half of the culture to induce thiol oxidative stress. Samples were removed before addition of diamide and at the indicated time points and β-galactosidase activity was determined. Cell lysates of the same samples were analyzed by SDS–PAGE and Western blot against Spx (lower panel). Representative data from at least two experiments are shown. **(B)** Same as **(A)** for strain BNM328, carrying a transcriptional *Phag*-*lacZ* fusion.

Whereas, the non-stressed control samples displayed a normal pattern of flagellar gene expression, *PflgB*-*lacZ* and *Phag*-*lacZ* activity strongly decreased for a period of 1–1.5 h after the application of oxidative stress (Figures 7A,B). Notably, Western blot analysis of the same samples with Spx-specific antibodies revealed that Spx protein was present in high amounts at the time points, at which flagellar gene expression was most strongly repressed (Figures 7A,B).

In summary, our data indicate that Spx acts as a negative regulator of the *flgB* promoter, which also affects σ^D^ levels and thus transcription from the *hag* promoter.

### Spx regulates motility indirectly on both *flgB* and *hag* promoter

Our results clearly demonstrate that a raised cellular level of Spx results in a repression of the *flgB* promoter. We already demonstrated that Spx does not act on this promoter via DegU (Figure S6). Furthermore, we tested whether the defect of swimming motility in a *clpX* mutant is suppressed by a *codY* mutation. Swimming motility, Hag protein levels as well as *flgB* promoter activity were not increased in the *clpX codY* double mutant compared to the *clpX* single mutant (Figures S7B–E).

Interestingly, the *hag* promoter activity was increased especially at later time points in the double *codY clpX* mutant strain. These data suggest that CodY might be somehow indirectly involved in the Spx dependent repression of the *hag* promoter in the *clpX* mutant. The results presented earlier in Figure S3 already suggested an influence of Spx on the *hag* promoter independent of its influence on the *flgB* promoter (Figure S3). However, Spx-mediated down-regulation of the *flgB* promoter appears to be mostly independent of *codY* under our experimental conditions and in our strain background (Figure S7).

To test whether Spx directly binds to the *flgB* promoter, we performed electrophoretic mobility shift assays using *flgB* promoter DNA fragments and purified Spx protein (see SectionMaterials and Methods). In accordance with previously published results on Spx (Nakano et al., 2010), we did not observe DNA binding of Spx to the P*flgB* promotor region (Figure S11), suggesting that Spx indirectly regulates P*flgB* promoter activity.

## Discussion

### Regulatory and general proteolysis in swimming motility of *B. subtilis*

In this work, we present a detailed analysis of the impact of Clp proteases on swimming motility in the model organism *B. subtilis*. We found that regulatory proteolysis of the transcription factors DegU and Spx by ClpCP and ClpXP, respectively, is an important mechanism to facilitate and control swimming motility. In the absence of these proteases, active DegU-P or Spx can accumulate and negatively regulate expression of the *fla/che* operon, resulting in low σ^D^ levels, lower expression of late flagellar genes and a loss of swimming motility. As already suggested for *B. subtilis* mutated in *clpC* (Rashid et al., 1996), these results may also explain the previously observed increased cell chaining in *clpP* and *clpX* mutants (Gerth et al., 1998; Msadek et al., 1998), since autolysin genes, such as *lytC* and *lytD*, which are required for cell separation during division, are controlled by σ^D^.

Regulation of flagellar assembly is also strongly influenced by regulatory proteolysis in *E. coli* or *Salmonella*, where the stability of the master regulator FlhDC and the flagellar sigma factor FliA are controlled by ClpXP (Tomoyasu et al., 2003; Barembruch and Hengge, 2007; Kitagawa et al., 2011; Takaya et al., 2012).

A master regulator and activator of motility, such as FlhDC in *E. coli* has not been identified in *B. subtilis* and the two proteins with a described activator function, SwrA (Kearns and Losick, 2005) and DegU (Tsukahara and Ogura, 2008), are both dispensable for normal expression of the *fla/che* operon for swimming motility in *B. subtilis* 168 strains. In most laboratory strains, such as *B. subtilis* 168, SwrA is encoded as a cryptic gene and not synthesized. In less domesticated *B. subtilis* strains, such as the biofilm forming NCIB 3610 *B. subtilis* strain, SwrA, like a small number of other regulatory proteins, is present and active as an activator of swimming motility even enabling swarming motility (Kearns et al., 2004; McLoon et al., 2011). It was suggested that SwrA acts in conjunction and interacting with DegU-P, switching it from a repressor to an activator of the *flgB* promoter (Ogura and Tsukahara, 2012; Mordini et al., 2013). Interestingly, when we tested the effect of *clpC* and *clpX* mutations on a *B. subtilis* 168 strain complemented with *swrA*^+^ and *degQ*^+^ alleles or the *B. subtilis* NCIB 3610 strain encoding SwrA, we still observed a negative effect on swimming motility (Figure S12).

### ClpCP influences motility by controlling the activity of DegU~P

We demonstrate here that ClpCP regulates swimming motility not only by proteolysis of ComK, as previously reported (Liu and Zuber, 1998), but also by controlling the activity or stability of DegU, which was recently identified as a proteolysis target of ClpCP (Ogura and Tsukahara, 2010). In accordance with these observations, we observed that both the *flgB* and *hag* promotors were down-regulated in the *clpC* mutant in a *degU*-dependent manner and the σ^D^ and Hag levels were strongly decreased, rendering the bacteria non-motile.

The function of DegU in motility development is complex and has been controversially discussed in the literature. Presumably, DegU can act both as an activator (Tsukahara and Ogura, 2008) and in its phosphorylated form as a repressor (Amati et al., 2004) of the *flgB* promoter. However, it has been demonstrated that *degU* is required for swarming motility, whereas the gene is dispensable for swimming motility (Kobayashi, 2007; Verhamme et al., 2007). Our data are consistent with this hypothesis, as *degSU* mutants were motile and producing Hag and the *degSU* mutation had only a minor effect on transcription of motility genes in our hands. Therefore, we conclude that under our conditions and in our strain background only the repressor function of DegU in its phosphorylated form is relevant for swimming motility and influenced by ClpC.

The *flgB* promoter features two DegU binding sites, one located upstream of the promoter (BR1) and one downstream in the *flgB* coding region (BR2; Tsukahara and Ogura, 2008). The results presented here suggest that the downstream BR2 binding site is required for repression of the *flgB* promoter by DegU, since the longer *lacZ* fusion that incorporates the BR2 site was down-regulated in the *clpC* mutant in a *degU*-dependent manner whereas the shorter fusion was not affected by the *clpC* mutation (Figure 2). These data are in accordance with those of Tsukahara et al., who reported that the BR2 binding site is required for *PflgB* repression in a *B. subtilis* strain carrying a *degU32* point mutant, which results in hyperphosphorylated DegU (Tsukahara and Ogura, 2008).

The long distance of the BR2 site from the core promoter suggests that DegU most probably does not repress *flgB* transcription by restricting access of RNA polymerase to the promoter. A similar mechanism was demonstrated for the repressor CodY, which binds to a site downstream of the *ybgE* transcription start and negatively regulates transcription elongation by a roadblock mechanism, resulting in a short terminated mRNA fragment (Belitsky and Sonenshein, 2011).

Interestingly, it was reported that phosphorylated DegU acts as a negative regulator of motility by transcriptional activation of *flgM* (Hsueh et al., 2011). This effect provides an additional explanation for the observed down-regulation of the *hag* promoter, which is σ^D^-dependent and therefore negatively regulated by FlgM, in the *clpC* mutant.

DegU has been described as a cellular rheostat that allows adequate expression of different groups of genes during transition to stationary phase to allow processes such as competence, biofilm formation, and motility. It was proposed that DegU degradation by ClpCP plays a part in fine-tuning of DegU auto-activation (Veening et al., 2008; Ogura and Tsukahara, 2010). The results presented here suggest that DegU activity and stability is also important for swimming motility. We assume that ClpCP-mediated inhibition and proteolysis ensures that active DegU concentration in the cell is kept below a threshold level, such that the *flgB* promoter is de-repressed, but high enough to allow expression of other DegU-activated genes.

Most studies suggest that DegU acts as a *PflgB* repressor primarily in its phosphorylated form (Verhamme et al., 2007; Tsukahara and Ogura, 2008) and the experiments of Ogura and colleagues suggest that only phosphorylated DegU is targeted for ClpCP-mediated degradation (Ogura and Tsukahara, 2010). However, we observed in our strain and growth conditions some elevated DegU levels *in vivo*, but not to the extent observed before. Nevertheless, our experimental data are consistent with the role of DegU~P as a repressor of motility, which is specifically inhibited by ClpC. It should be noted that ClpC alone could be sufficient to repress DegU-P activity by unfolding DegU-P without targeting it to ClpP. It would be very interesting to explore by what mechanism phosphorylated DegU is recognized by ClpC and under what conditions it is targeted for degradation to ClpCP.

### Regulatory proteolysis of Spx by ClpXP influences swimming motility

The second principal finding of this paper is the observation that swimming motility is inhibited in a *clpX* mutant via the stabilization of the ClpXP substrate Spx, which acts as a negative regulator of motility. We have demonstrated that an *spx* mutant suppresses the decreased swimming motility observed in a *clpX* mutant (Figure 4) and that raised levels of Spx^DD^ in a *clpX*^+^ background inhibit motility (Figure 5). Our results suggest that Spx inhibits motility at the level of the *flgB* promoter (Figures 5, 6) and can in addition also influence the *hag* promoter (Figure S3). Furthermore, we could demonstrate that motility gene expression is transiently inhibited during thiol oxidative stress, which activates and stabilizes Spx (Figure 7).

Spx-mediated down-regulation of motility provides a connection between motility and a stress response pathway. The oxidative stress response requires a restructuring of the proteome redox enzymes and chaperones (Zuber, 2009). Likewise, swimming motility requires a substantial effort both for production of the flagellar proteins and their assembly (Chevance and Hughes, 2008). Exerting both programs at the same time could be detrimental for these cells. Our observations suggest that upon oxidative stress or induction of Spx^DD^ the cells give priority to the stress response. However, they can resume motility development after oxidative stress has been alleviated (Figures 6, 7).

Importantly, this does not necessarily require that individual cells are non-motile during the stress response, since only flagellar gene expression is down-regulated. Already existing flagella could continue to function, which might even be an advantage for cells, enabling them to escape from the source of the stress by chemotaxis and swimming motility.

### Possible mechanisms of motility regulation by Spx

Spx is a transcription factor, which interacts with the alpha subunit of RNA polymerase and enhances polymerase binding to certain promoters (Reyes and Zuber, 2008; Nakano et al., 2010). This mechanism is shared by a number of transcriptional activators, including response regulators such as ComA (Nakano et al., 2003b). According to the interference model, Spx does not directly act as a repressor, but restricts access of other transcription factors to the RNA polymerase alpha subunit when present at high concentrations. By this mechanism for example are competence genes repressed in the presence of Spx, because phosphorylated ComA can no longer bind to RNA polymerase (Nakano et al., 2003b). As already mentioned, an activator of transcription of the *flgB* promoter in *B. subtilis* 168 is not known, therefore it is very unlikely that Spx could act as a repressor of P*flgB* by interfering with an activator.

It was recently observed that Spx can activate the transcription of *degSU* (Shiwa et al., 2015), which could potentially effect the regulation of motility. However, we observed that a deletion of *degSU* did not interfere with the Spx mediated inhibition of motility (Figure S6) and no elevated DegU levels were observed in a *clpX* mutant (Figure 2G). However, the implications of raised levels of DegU and DegS on regulation of motility should be investigated in more detail.

DNA binding of Spx in the absence of RNA polymerase alpha CTD has never been observed and we have shown that Spx does not bind to the *flgB* promoter fragment *in vitro* (Figure S11). An indirect regulation of motility by Spx is also supported by a study, in which the Spx regulon was analyzed by tiling arrays and a genome wide characterization of Spx binding sites was accomplished. Spx-dependent repression of a number of motility and chemotaxis genes was observed in these experiments, but since no relevant Spx binding sites e.g., near the *flgB* and *hag* promoter were identified, this was considered an indirect Spx-mediated effect (Rochat et al., 2012). This suggests that Spx rather indirectly influences the *flgB* promoter and *fla/che* expression, for example by transcriptional activation of a repressor or other not yet identified intracellular signal transduction mechanisms. More experiments will be necessary to understand and elucidate the mechanism by which Spx influences motility via the *flgB* and *hag* promoters in *B. subtilis*.

In summary, we have uncovered two additional pathways, by which regulatory proteolysis affects swimming motility in *B. subtilis*. We could demonstrate that ClpCP contributes to motility development by controlling the stability of the response regulator DegU, which can act as a repressor of the *fla/che* operon. In turn, ClpXP facilitates swimming motility by proteolysis of its substrate Spx. We could show that the oxidative stress regulator Spx acts as a negative regulator of motility on the *flgB* promoter and the *hag* promoter. The additionally observed Spx-mediated repression of *hag* might also be facilitated by a yet unknown posttranscriptional process. Importantly, the influence of Spx on motility could also be observed in wild type cells during oxidative stress and can therefore be considered as a biologically relevant stress response mechanism.

These results highlight the complex involvement of controlled proteolysis in the regulation of motility and its intricate connections to stress response pathways such as the Spx controlled thiol stress response (Zuber, 2004, 2009) or heat shock response (Runde et al., 2014) and the various processes (such as e.g., biofilm formation) controlled by the master regulator DegU (Murray et al., 2009).

## Author contributions

NM designed research, performed experiments, analyzed results, and wrote the paper. JH designed research, performed experiments and analyzed data. HS designed research, performed experiments and analyzed data KT designed research, analyzed results, and wrote the paper.

## Funding

We acknowledge support by Deutsche Forschungsgemainschaft (Tu106/6, Tu106/7) and Open Access Publishing Fund of Leibniz Universität Hannover.

### Conflict of interest statement

The authors declare that the research was conducted in the absence of any commercial or financial relationships that could be construed as a potential conflict of interest.
