# Supplementary material for: Role of Hsp100/Clp Protease Complexes in Controlling the Regulation of Motility in Bacillus subtilis
Source: Front Microbiol. 2016 Mar 16;7:315. doi: 10.3389/fmicb.2016.00315 (PMC4793158; doi:10.3389/fmicb.2016.00315)
Supplement: Supplementary file 1 [file DataSheet1.pdf]

Supplementary Tables

Table S1: List of strains

Strains	Relevant genotype/properties	Source/construction
B.subtilis 168	*trpC2*	(Anagnostopoulos and Spizizen, 1961)
BUG1	*trpC2 ΔclpP::spec*	(Gerth et al., 2004)
BNM103	*trpC2 ΔclpP::spec*	BUG1 → *B. subtilis* 168
BNM104	*trpC2 ΔclpP::spec*	Suppressor mutant obtained from BNM103
QBP418	PY79 *ΔclpC::tet*	(Pan et al., 2001)
BNM105	*trpC2 ΔclpC::tet*	QBP418 → *B. subtilis* 168
BMM103	*trpC2 ΔclpE::spec*	(Miethke et al., 2006)
BNM106	*trpC2 ΔclpE::spec*	BMM103→ *B. subtilis* 168
BEK90	*trpC2 ΔclpX::kan*	(Gerth et al., 2004)
BNM107	*trpC2 ΔclpX::kan*	BEK90 → *B. subtilis* 168
ORB3834	*trpC2 pheA1 Δspx::kan*	(Nakano et al., 2001)
BNM109	*trpC2 ΔclpX::tet*	This work
BNM111	*trpC2 Δspx::kan*	ORB3834 → *B. subtilis* 168
ORB3838	*trpC2 pheA1 Δspx::kan ΔclpX::spec*	(Nakano et al., 2001)
BNM112	*Δspx::kan ΔclpX::spec*	ORB3838 → *B. subtilis* 168
BNM126	*trpC2 Δhag*	pMAD*hag* →*B. subtilis* 168
NRS1499	NCIB 3610 *ΔdegSU::spec*	N. Stanley-Wall, unpublished
BNM138	*trpC2 ΔdegSU::spec*	NRS1499 → *B. subtilis* 168

BNM140	*trpC2 ΔdegSU::spec ΔclpC::tet*	BNM105/BNM138 → *B. subtilis* 168
BNM142	*trpC2 ΔdegSU::spec ΔclpX::kan*	BNM107/BNM138→ *B. subtilis* 168
PS258	*trpC2 ΔcodY::erm*	(Brinsmade and Sonenshein, 2011)
BNM143	*trpC2 ΔcodY::erm*	PS258 → *B. subtilis* 168
BNM147	*trpC2 ΔcodY::erm ΔclpX::kan*	BNM107 → BNM143
BNM149	*trpC2 ΔcomK*	pMAD*comK* → *B. subtilis* 168
BNM150	*trpC2 ΔcomK ΔclpC::tet*	BNM105 → BNM149
DS791	NCIB3610 *amyE::PflgB–lacZ cat*	(Kearns and Losick, 2005)
BNM301	*trpC2 amyE::PflgB–lacZ cat*	DS791 → *B. subtilis* 168
BNM302	*trpC2 amyE::PflgB–lacZ cat ΔclpP::spec*	BNM103 → BNM301
BNM303	*trpC2 amyE::PflgB–lacZ cat ΔclpC::tet*	BNM105 → BNM301
BNM305	*trpC2 amyE::PflgB–lacZ cat ΔclpX::kan*	BNM107 → BNM301
BNM306	*trpC2 amyE::PflgB–lacZ cat ΔdegSU::spec*	BNM138 → BNM301
BNM307	*trpC2 amyE::PflgB–lacZ cat Δspx::kan*	BNM111 → BNM301
BNM308	*trpC2 amyE::PflgB–lacZ cat Δspx::kan ΔclpX::spec*	BNM112 → BNM301
BNM309	*trpC2 amyE::PflgB–lacZ cat ΔcodY::erm*	BNM143 → BNM301
DS793	NCIB3610 *amyE::Phag–lacZ cat*	(Kearns and Losick, 2005)
BNM328	*trpC2 amyE::Phag–lacZ cat*	DS793 → *B. subtilis* 168
BNM329	*trpC2 amyE::Phag–lacZ cat ΔclpP::spec*	BNM103 → BNM328
BNM330	*trpC2 amyE::Phag–lacZ cat ΔclpC::tet*	BNM105 → BNM328
BNM332	*trpC2 amyE::Phag–lacZ cat ΔclpX::kan*	BNM107 → BNM328

BNM333	*trpC2 amyE::Phag-lacZ cat ΔdegSU::spec*	BNM138 → BNM328
BNM334	*trpC2 amyE::Phag–lacZ cat Δspx::kan*	BNM111 → BNM328
BNM335	*trpC2 amyE::Phag–lacZ cat Δspx::kan ΔclpX::spec*	BNM112 → BNM328
BNM336	*trpC2 amyE::Phag–lacZ cat ΔcodY::erm*	BNM143 → BNM328
BNM338	*trpC2 amyE::Phag–lacZ cat ΔdegSU::spec ΔclpC::tet*	BNM138 → BNM328
BNM339	*trpC2 amyE::Phag–lacZ cat ΔdegSU::spec ΔclpX::kan*	BNM138/BNM107 → BNM328
BNM341	*trpC2 amyE::PflgB–lacZ cat ΔdegSU::spec ΔclpC::tet*	BNM138/BNM105 → BNM301
BNM343	*trpC2 amyE::Phag–lacZ cat ΔcodY::erm ΔclpX::kan*	BNM107 → BNM336
BNM346	*trpC2 amyE::flgB152p–lacZ cat*	p*flgB152* → *B. subtilis* 168
BNM347	*trpC2 amyE::flgB152p–lacZ cat ΔdegSU::spec*	BNM138 → BNM346
BNM348	*trpC2 amyE::flgB152p–lacZ cat ΔclpC::tet*	BNM105 → BNM346
BNM349	*trpC2 amyE::flgB152p–lacZ cat ΔdegSU::spec ΔclpC::tet*	BNM105/BNM138 → BNM346
BNM350	*trpC2 Δspx::kan amyE::*$P_{hyperspank(Hy)}$*–spx spec*	pMMN521 → *B .subtilis* 168 (Nakano et al., 2003)
BNM351	*trpC2 Δspx::kan amyE::*$P_{hyperspank(Hy)}$*–*spx^{DD} *spec*	pSN56 → BNM111 (Nakano et al., 2003)
BNM421	*trpC2 Δhag amyE::*P_{xyl}*-hag1 cat*	pX*hag1* → BNM126
BNM422	*trpC2 Δhag amyE::*P_{xyl}*–hag1 cat ΔclpP::spec*	BNM103 → BNM421
BNM423	*trpC2 Δhag amyE::*P_{xyl}*–hag1 cat ΔclpC::tet*	BNM105 → BNM421
BNM424	*trpC2 Δhag amyE::*P_{xyl}*–hag1 cat ΔclpE::spec*	BNM106 → BNM421
BNM425	*trpC2 Δhag amyE::*P_{xyl}*–hag1 cat ΔclpX::kan*	BNM107 → BNM421
BNM426	*trpC2 Δhag amyE::*P_{xyl}*–hag4 cat*	pX*hag4* → BNM126

BNM427	*trpC2 Δhag amyE::*P_{xyl}*–hag4 cat ΔclpP::spec*	BNM103 → BNM426
BNM428	*trpC2 Δhag amyE::*P_{xyl}*–hag4 cat ΔclpC::tet*	BNM105 → BNM426
BNM429	*trpC2 Δhag amyE::*P_{xyl}*–hag4 cat ΔclpE::spec*	BNM106 → BNM426
BNM430	*trpC2 Δhag amyE::*P_{xyl}*–hag4 cat ΔclpX::kan*	BNM107 → BNM426
BNM436	*trpC2 Δhag amyE::*P_{xyl}*–hag4 cat Δspx::kan*	BNM111 → BNM426
BNM437	*trpC2 Δhag amyE::*P_{xyl}*–hag4 cat Δspx::kan ΔclpX::spec*	BNM112 → BNM426
BNM810	*trpC2 amyE::*$P_{hyperspank(Hy)}$*–*spx^{DD} *spec*	pSN56 → BNM101 (Nakano et al., 2003)
BNM834	*trpC2 amyE::PflgB–lacZ cat ΔcodY::erm ΔclpX::kan*	BNM107 → BNM309
BNM840	*trpC2 ΔcodY::erm ΔclpX::kan*	BNM107 → BNM143
BNM844	*trpC2 amyE::*$P_{hyperspank(Hy)}$*–*spx^{DD} *spec ΔcodY::erm*	BNM143 → BNM810
LUW272	*trpC2 ΔyjbH::spec*	(Rogstam et al., 2007)
BNM855	*trpC2 ΔyjbH::spec*	LUW272 → BNM101
BNM857	*trpC2 ΔyjbH::spec Δspx::kan*	BNM855 → BNM111
ABH282	PY79 *ywrK::Tn917::amyE::cat*	pAH120 → *B. subtilis* PY79 (Camp and Losick, 2009)
BNM860	*trpC2 ywrK::Tn917::amyE::cat*	ABH282 → BNM101
BNM866	*trpC2 ywrK::Tn917::amyE::* $P_{hyperspank(Hy)}$*–*spx^{DD} *spec*	pSN56 → BNM860
BNM878	*trpC2 ywrK::Tn917::amyE::* $P_{hyperspank(Hy)}$*–*spx^{DD} *spec amyE::PflgB–lacZ cat*	BNM301 → BNM866
BNM1001	*trpC2 ywrK::Tn917::amyE::* $P_{hyperspank(Hy)}$*–*spx^{DD} *spec amyE::Phag–lacZ cat*	BNM328 → BNM866
swrA^{+} *degQ*$^{+}$	*trpC2 sacA::swrA cat thrC::degQ kan*	Gift of Nicola Stanley-Wall Jörn Hoßmann, Diploma thesis
BNM1266	*trpC2 sacA::swrA cat thrC::degQ kan ΔclpC::tet*	BNM105→ *swrA*$^{+}$ *degQ*$^{+}$

BNM1268	*trpC2 sacA::swrA cat thrC::degQ kan ΔclpP::spec*	BNM103→ *swrA*$^{+}$ *degQ*$^{+}$
BNM1270	*trpC2 sacA::swrA cat thrC::degQ kan ΔclpX::tet*	BNM109→ *swrA*$^{+}$ *degQ*$^{+}$
DK1042	NCIB3610 *comIQ12L*	BGSC (Lab of Dan Kearns)
BHS122	NCIB3610 *comIQ12L ΔclpC::tet*	BNM105→ DK1042
BHS123	NCIB3610 *comI-Q12L ΔclpX::kan*	BNM107→ DK1042
E.coli XL1-blue competent cells	*recA1 endA1 gyrA96 thi-1 hsdR17 supE44 relA1 lac [F proAB lacI*^{q} *ZM15 Tn10 (Tet*^{r}*)]*	Stratagene
E.coli FI1202	*lacI*^{q} *lacL8 gln5::Tn5 1202*	(Fiedler and Weiss, 1995)

Table S2: List of plasmids

**Plasmid**	**Construction**	**Reference**
pQE60	C-terminal His_6-tag fusion	Qiagen
pQE60–*hag*	*hag* in *Nco*I/*Bam*HI of pQE60	This work
pQE60–*spx*	*spx* in *Nco*I/*Bam*HI of pQE60	This work
pDG268	Vector for transcriptional *lacZ* fusion	(Antoniewski et al., 1990)
p*flgB152*	*flgB152* fragment in *Eco*RI/*Bam*HI of pDG268	This work
pMAD	Vector for markerless deletions	(Arnaud et al., 2004)
pMAD–*hag*	*hag* flanking regions in *Bam*HI/*Nco*I of pMAD	(Blair et al., 2008)
pMAD–*comK*	*comK* flanking regions in *Bam*HI/*Nco*I of pMAD	This work
pMMN521	$P_{hyperspank(Hy)}$–*spx* for *amyE* insertion	(Nakano et al., 2003)
pSN56	$P_{hyperspank(Hy)}$–*spx*^{DD} for *amyE* insertion	(Nakano et al., 2003)
pX	Expression from P_{xyl} in *amyE*	(Kim et al., 1996)
pX–*hag1*	*hag1* fragment in *Bam*HI of pX	This work

pX–*hag4*	*hag4* fragment in *Bam*HI of pX	This work

References

Anagnostopoulos, C., and Spizizen, J. (1961). Requirements for Transformation in *Bacillus subtilis*. *J. Bacteriol.* 81, 741–746.

Antoniewski, C., Savelli, B., and Stragier, P. (1990). The *spoIIJ* gene, which regulates early developmental steps in *Bacillus subtilis*, belongs to a class of environmentally responsive genes. *J. Bacteriol.* 172, 86–93.

Arnaud, M., Chastanet, A., and Débarbouillé, M. (2004). New vector for efficient allelic replacement in naturally nontransformable, low-GC-content, gram-positive bacteria. *Appl. Environ. Microbiol.* 70, 6887–6891. doi:10.1128/AEM.70.11.6887-6891.2004.

Blair, K. M., Turner, L., Winkelman, J. T., Berg, H. C., and Kearns, D. B. (2008). A molecular clutch disables flagella in the *Bacillus subtilis* biofilm. *Science* 320, 1636–1638. doi:10.1126/science.1157877.

Brinsmade, S. R., and Sonenshein, A. L. (2011). Dissecting complex metabolic integration provides direct genetic evidence for CodY activation by guanine nucleotides. *J. Bacteriol.* 193, 5637–5648. doi:10.1128/JB.05510-11.

Camp, A. H., and Losick, R. (2009). A feeding tube model for activation of a cell-specific transcription factor during sporulation in *Bacillus subtilis*. *Genes Dev.* 23, 1014–1024. doi:10.1101/gad.1781709.

Fiedler, U., and Weiss, V. (1995). A common switch in activation of the response regulators NtrC and PhoB: phosphorylation induces dimerization of the receiver modules. *EMBO J.* 14, 3696–3705.

Gerth, U., Kirstein, J., Mostertz, J., Waldminghaus, T., Miethke, M., Kock, H., et al. (2004). Fine-tuning in regulation of Clp protein content in *Bacillus subtilis*. *J. Bacteriol.* 186, 179–191.

Kearns, D. B., and Losick, R. (2005). Cell population heterogeneity during growth of *Bacillus subtilis*. *Genes Dev.* 19, 3083–3094. doi:10.1101/gad.1373905.

Kim, L., Mogk, A., and Schumann, W. (1996). A xylose-inducible *Bacillus subtilis* integration vector and its application. *Gene* 181, 71–76.

Miethke, M., Hecker, M., and Gerth, U. (2006). Involvement of *Bacillus subtilis* ClpE in CtsR degradation and protein quality control. *J. Bacteriol.* 188, 4610–4619. doi:10.1128/JB.00287-06.

Nakano, M. M., Hajarizadeh, F., Zhu, Y., and Zuber, P. (2001). Loss-of-function mutations in *yjbD* result in ClpX- and ClpP-independent competence development of *Bacillus subtilis*. *Mol. Microbiol.* 42, 383–394.

Nakano, S., Küster-Schöck, E., Grossman, A. D., and Zuber, P. (2003). Spx-dependent global transcriptional control is induced by thiol-specific oxidative stress in *Bacillus subtilis*. *Proc. Natl. Acad. Sci. U. S. A.* 100, 13603–13608. doi:10.1073/pnas.2235180100.

Pan, Q., Garsin, D. A., and Losick, R. (2001). Self-reinforcing activation of a cell-specific transcription factor by proteolysis of an anti-sigma factor in *B. subtilis*. *Mol. Cell* 8, 873–883.

Rogstam, A., Larsson, J. T., Kjelgaard, P., and von Wachenfeldt, C. (2007). Mechanisms of adaptation to nitrosative stress in *Bacillus subtilis*. *J. Bacteriol.* 189, 3063–3071. doi:10.1128/JB.01782-06.

Table S3: List of primers

Primer	Sequence
PhagQE60–for	catg**ccatgg**tgagaattaaccacaatattgcagcg
PhagQE60–rev	gg**ggatcc**acgtaataattgaagtacgttttgc
spxpQE60–for	ggagtgaagat**ccatgg**ttacac
spxpQE60–rev	gatacgat**ggatcc**gtttgccaaacgc
hag1–for	ccc**ggatcc**atgagaattaaccacaatattgc
hag1–rev	ccc**ggatcc**ttaacgtaataattgaagtacgttttgc
hag4–for	ccc**ggatcc**tgtagccgggaggaggcgca
hag4–rev	cccc**ggatcc**agaccctggcaacgccaagg
flgB152–for	g**gaattc**ttgctgaccgtgtcggcattac
flgB152–rev	cg**ggatcc**gcgatattattagttatgac
flgB (-209 to -6)–for	ggaattcattttgcatttttcttcaaaaag
flgB (-209 to -6)–rev	cgggatcctattgtaagaaataacaggc
flgB (-106 to 98)–for	ggaattcctaacaatctaggactttatac
flgB (-106 to 98)–rev	cgggatcccaagattttgtatcgt
flgB (-1 to 203)–for	ggaattctatagttttacaattctcgac
flgB (-1 to 203)–rev	cgggatccaattttggaaagagactttttttg
hag–probe–for	attgaaagttgttgatgaag
clpXtetP1	agtgacattccctgaggag
clpXtetP2	gaacaacctgcattgcaagatctttcaccccttaatcttg
clpXtetP3	ttgatcctttttttataacaggaattcagataagcacaaacct cctg
clpXtetP4	tttggcattctcgtggtcgcg

Supplementary Figure Legends

Fig S1 in vivo stability of Hag
B. subtilis wild type cells were grown in Belitsky minimal medium and pulse labeled with 25 µCi ^{35}S–methionine for 10 minutes. Samples were TCA–precipitated and immunoprecipitation was performed with Hag–specific antibodies and protein A–coated magnetic beads. Samples were separated by SDS–PAGE (12.5 %) and subjected to autoradiography.

Fig S2 Northern blot analysis of flagellar transcripts in wild type and *clp* mutant cells.
Northern blot using total RNA of the wild type and strains BNM126 (Δ*hag*), BNM103 (Δ*clpP*), BNM105 (Δ*clpC*), BNM106 (Δ*clpE*) and BNM107 (Δ*clpX*) grown to OD_{600} 1.0 at 37 °C in LB medium. 200 ng total RNA were separated by gel electrophoresis and blotted on nylon membranes. The RNA was hybridized to a digoxygenin (DIG)–labeled DNA probe against *hag*, and developed using anti–digoxygenin antibodies conjugated to alkaline phosphatase. Antibodies were detected using CDP–Star (Roche Applied Sciences). M: 100 ng DIG–labeled RNA Molecular Weight Marker III (Roche Applied Sciences).

Fig S3 Uncoupling *hag* expression from flagellar regulation.
(A) Hag Western blot analysis of strain BNM421 (WT), BNM422 (Δ*clpP*), BNM423 (Δ*clpC*), BNM424 (Δ*clpE*) and BNM425 (Δ*clpX*), carrying a xylose–inducible copy of *hag* at the *amyE* locus. Cells were grown to OD 1.0 at 37 °C in LB medium with 2 % w/v xylose (+) or in the absence of xylose (–). Cells were lysed by lysozyme treatment and boiling in SDS sample buffer (see Materials and Methods). (B) Same as A for strains BNM426 (WT), BNM427 (Δ*clpP*), BNM428 (Δ*clpC*), BNM429 (Δ*clpE*) and BNM430 (Δ*clpX*), in which the *hag* coding region along with the untranslated regions is expressed under the control of the P_{xyl} promoter. (C) Motility assay of strains BNM426 (WT), BNM427 (Δ*clpP*), BNM428 (Δ*clpC*), BNM429 (Δ*clpE*) and BNM430 (Δ*clpX*) in the absence (left) or presence (right) of 2 % w/v xylose. (D) Hag Western blots of strains BNM426 (WT), BNM430 (Δ*clpX*), BNM436 (Δ*spx*) and BNM437 (Δ*spx* Δ*clpX*), induced with 2 % w/v xylose.

Fig S4 *clpC* affects swimming motility and Hag levels via *comK*.
(A) Motility assay of *B. subtilis* wild type and Δ*clpC::tet* (BNM105), Δ*comK* (BNM149), and Δ*clpC::tet* Δ*comK* (BNM150). (B) Cells of the indicated strains (as in A) were grown to OD_{600} 1.0 at 37 °C. Cell lysates were analyzed by SDS–PAGE and Western blotting using anti–Hag antibodies.

Fig S5 DegU levels in wild type and *clp* mutant cells.
The wild type and strains BNM103 (Δ*clpP*), BNM105 (Δ*clpC*), BNM106 (Δ*clpE*), BNM107 (Δ*clpX*) and BNM138 (Δ*degSU*) were grown in LB medium at 37 °C to T–1 (one hour prior to entry into stationary phase, A), T0 (time of entry into stationary phase, B), T2 (C), T3 (D) and T4 (E), protoplasts were prepared and lysed (see Materials &

Methods). Lysates were separated by SDS–PAGE and subjected to Western blot analysis using polyclonal DegU antibodies (upper panels) or HtpG antibodies as a loading control (lower panels). Band intensities were normalized by calculating the ratio between the DegU signal and the HtpG signal and subsequently normalized to wild type levels of each time point.

Fig S6 The influence of *clpX* acts on motility independently of *degU*.
(A) Motility assay of the wild type and strains BNM107 (ΔclpX), BNM138 (Δ*degSU*), and BNM140 (Δ*degSU* Δ*clpX*). (B) β–galactosidase assay of the indicated strains carrying a *Phag–lacZ* fusion. Circles: wild type 168, triangles: Δ*clpX::kan* (BNM305), diamonds: Δ*degSU::spec* (BNM333), inverted triangles: Δ*degSU::spec* Δ*clpX::kan* (BNM339). Results from one representative experiment are depicted.

Fig S7 Clp proteases influence motility independently of *codY*.
(A) CodY Western blot of lysates from the wild type and strains BNM103 (Δ*clpP*), BNM105 (Δ*clpC*), BNM106 (Δ*clpE*) and BNM107 (Δ*clpX*) grown to OD_{600} 1.0 at 37 °C in LB medium. (B) Motility assay of the wild type and strains BNM107 (Δ*clpX::kan*), BNM143 (Δ*codY::erm*) and BNM147 (Δ*codY::erm* Δ*clpX::kan*). (C) Hag Western blot analysis, strains as in (A). (D) β–galactosidase assays of strains carrying a *PflgB–lacZ* fusion. Δ*clpX::kan* (BNM305, triangles), Δ*codY::erm* (BNM309, diamonds) and Δ*codY::erm* Δ*clpX::kan* (BNM834, inverted triangles). Results from one representative experiment are shown. (E) Same as (D) for the *Phag–lacZ* fusion. Circles: wild type 168 (BNM328), triangles: Δ*codY::erm* (BNM336) diamonds: Δ*clpX::kan* (BNM332), inverted triangles: Δ*codY::erm* Δ*clpX::kan* (BNM343).

FigG S8 A *clpP* suppressor mutant restores motility and Hag levels.
(A) Growth curves in LB medium at 37 °C of the *B. subtilis* wild type (circles), freshly transformed Δ*clpP*::spec mutant (BNM103, squares) and from a Δ*clpP::spec* suppressor mutant (BNM104, triangles). (B) Motility assay of the indicated strains (as in A). (C) Hag Western blot of the wild type and strain BNM104 (Δ*clpP* sup, upper panel) and Spx Western blot of the wild type, strain BNM104 (Δ*clpP* sup), and strain BNM103 (Δ*clpP*::spec).

Fig S9 Production of Spx^{DD} reduces *hag* mRNA levels and Hag protein levels.
(A) Northern blot analysis of samples from strain BNM351 (Δ*spx::kan amyE::*P_{Hy}–spx^{DD}) induced with 1 mM IPTG as indicated. Blots were hybridized with DIG–labeled *hag* probe. (B) Western blot analysis of samples from strains BNM350 (Δ*spx::kan amyE::*P_{Hy}–*spx*) and BNM351 (Δ*spx::kan amyE::*P_{Hy}–spx^{DD}) induced with 1 mM IPTG using Spx and Hag antibodies.

Fig S10 YjbH influences swimming motility and Hag levels.
(A) Motility assay of the wild type and strains BNM111 (Δ*spx*), BNM855 (Δ*yjbH*) and BNM857 (Δ*spx* Δ*yjbH*). (B) Hag Western blot analysis. Strains as in (A).

Fig S11 Electrophoretic mobility shift assay using purified Spx and *fla/che* promoter DNA fragments.

Gelshift experiments using different *PflgB* fragments (A: -209 to -6, B: -106 to 98, C: -1 to 203 relative to the *flgB* transcription start site) and purified Spx–His_6 (0, 1.25, 2.5 or 5 µM) were performed in the presence of poly–d–IC under oxidizing (1 mM diamide, left) and reducing conditions (5 mM DTT, right).

Fig S12 Influence of *clpC and clpX* mutations on swimming motility of *B. subtilis* strains carrying the *swrA*$^+$ and *degQ*$^+$ alleles. (A) Swimming plate with the indicated complemented *B. subtilis* 168 strains. (The plate was inoculated with 3 µl of a growing culture (OD_{600} of 1) and incubated for 7 h at 37 °C and o/n at room temperature) (B) A Western Blot (with anti Hag and anti HtpG serum) of extracts prepared from indicated *B. subtilis* strains (OD_{600} 0,9-1,1) 5 µg total protein was loaded. (C) Swimming plate of the wild type NCIB 3610 strain and the respective *clpC* and *clpX* mutant.
