# Supplementary figures and images for: Role of Hsp100/Clp Protease Complexes in Controlling the Regulation of Motility in Bacillus subtilis
Source: Front Microbiol. 2016 Mar 16;7:315. doi: 10.3389/fmicb.2016.00315 (PMC4793158; doi:10.3389/fmicb.2016.00315)

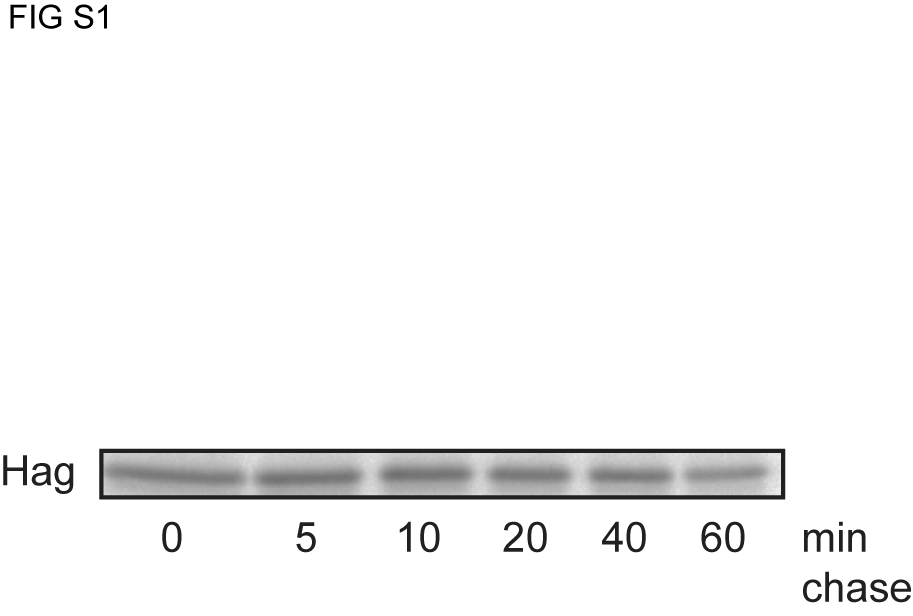

Supplement: Supplementary file 2 [file Image1.TIF]

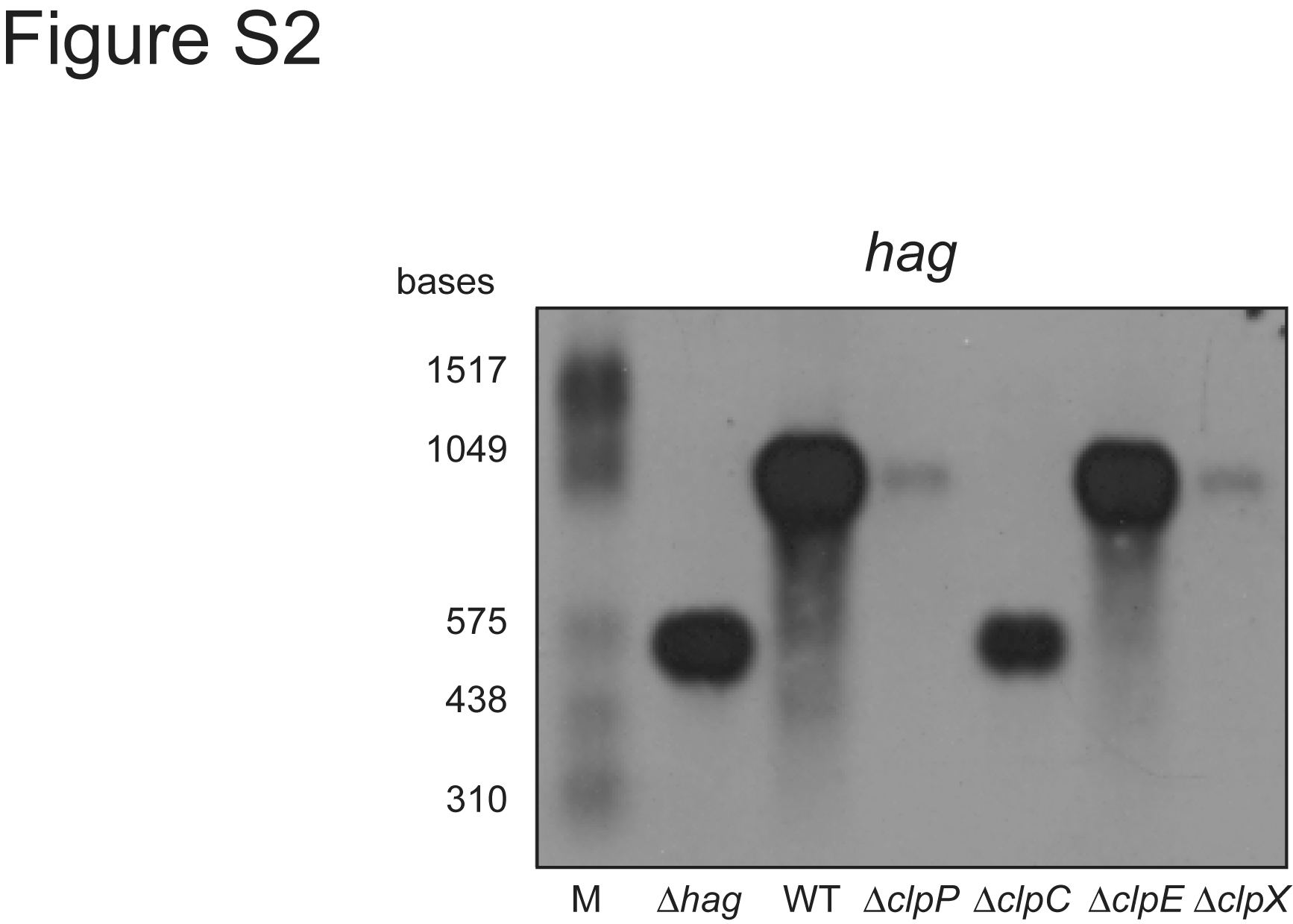

Supplement: Supplementary file 3 [file Image2.TIF]

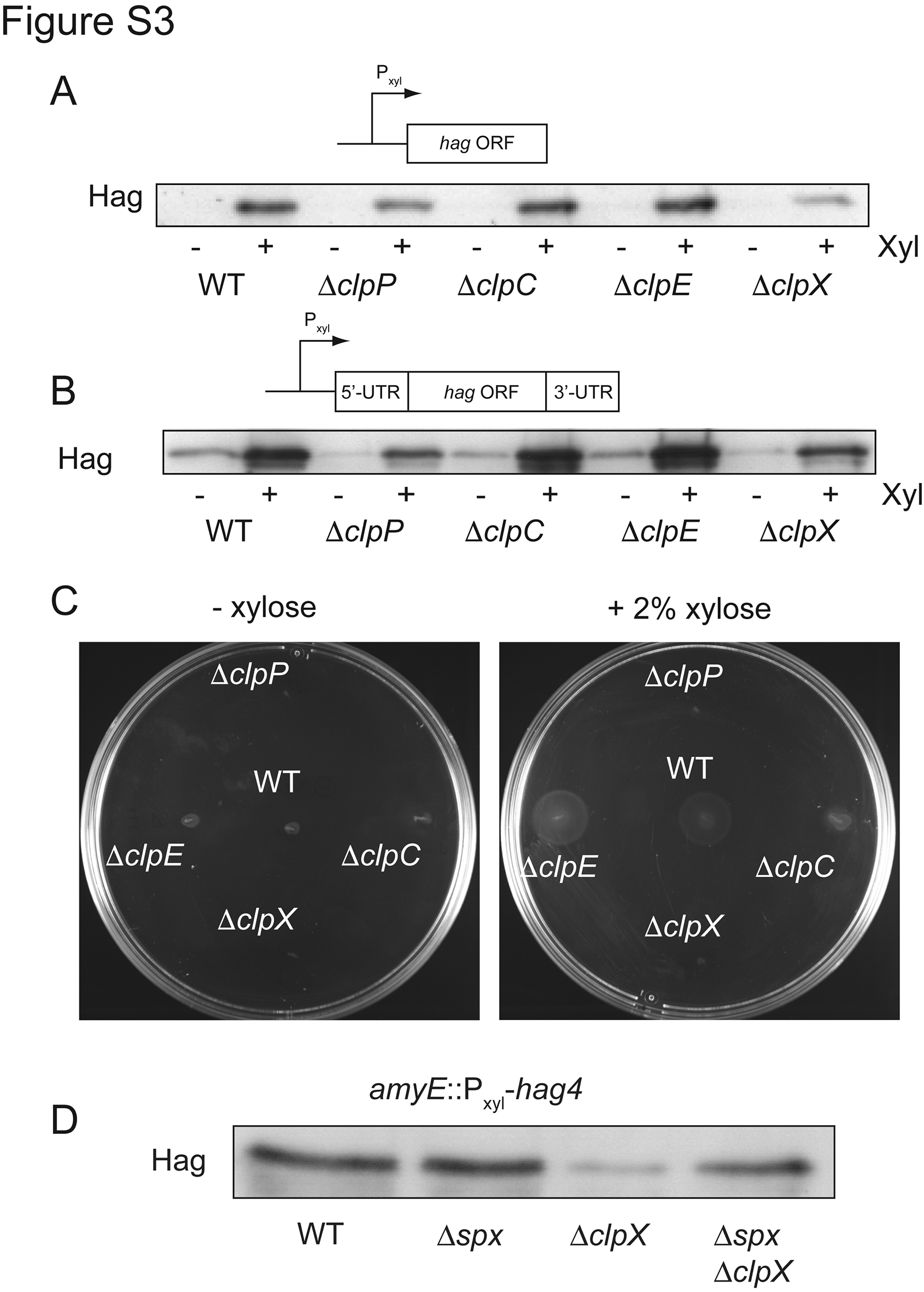

Supplement: Supplementary file 4 [file Image3.TIF]

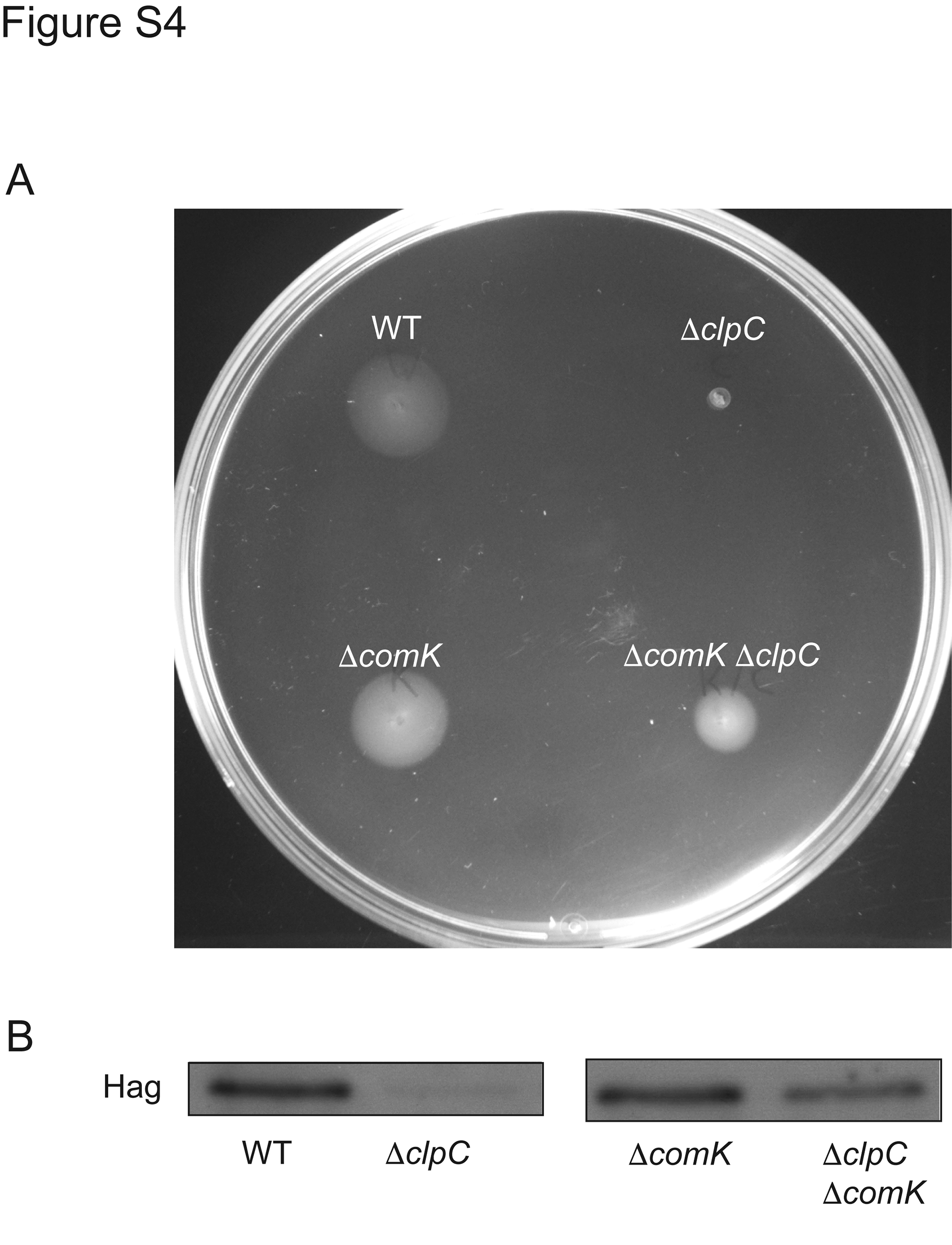

Supplement: Supplementary file 5 [file Image4.TIF]

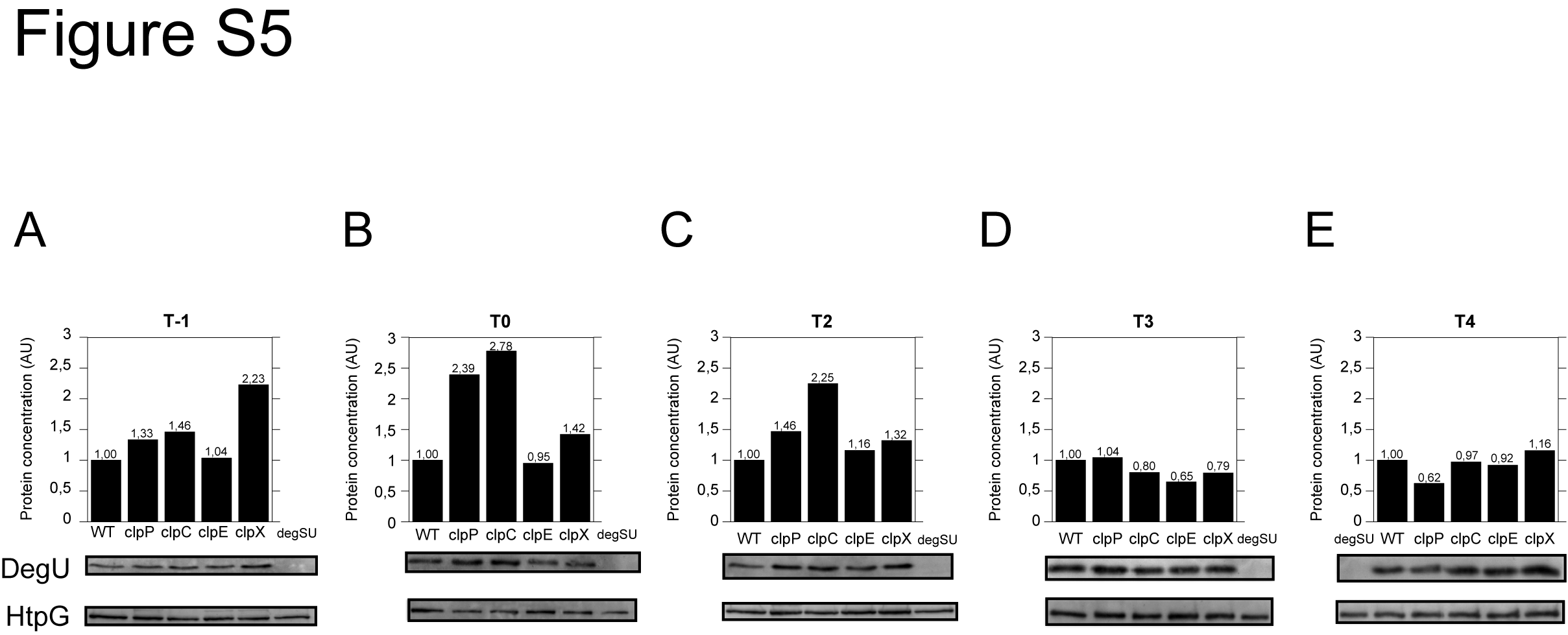

Supplement: Supplementary file 6 [file Image5.TIF]

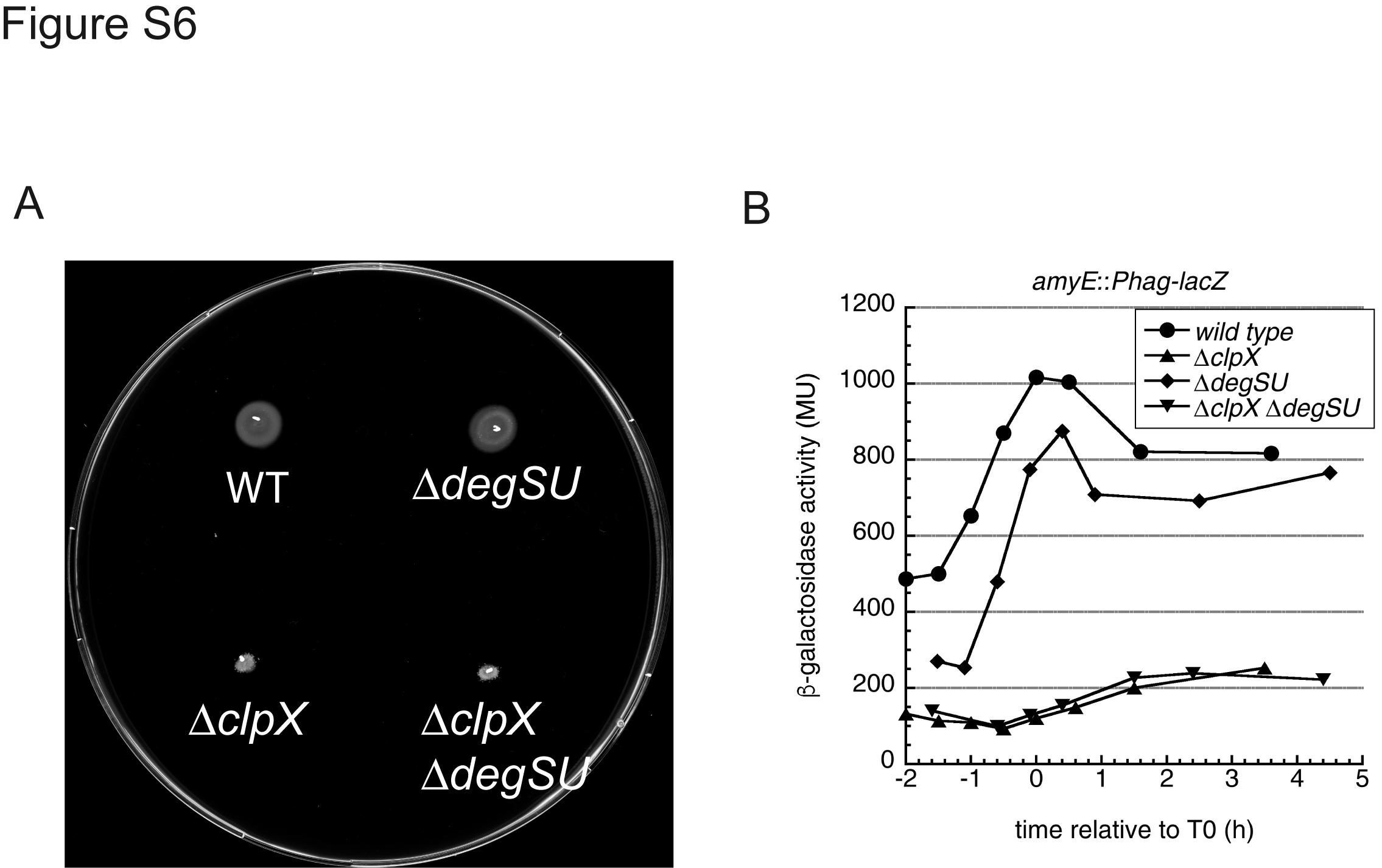

Supplement: Supplementary file 7 [file Image6.TIF]

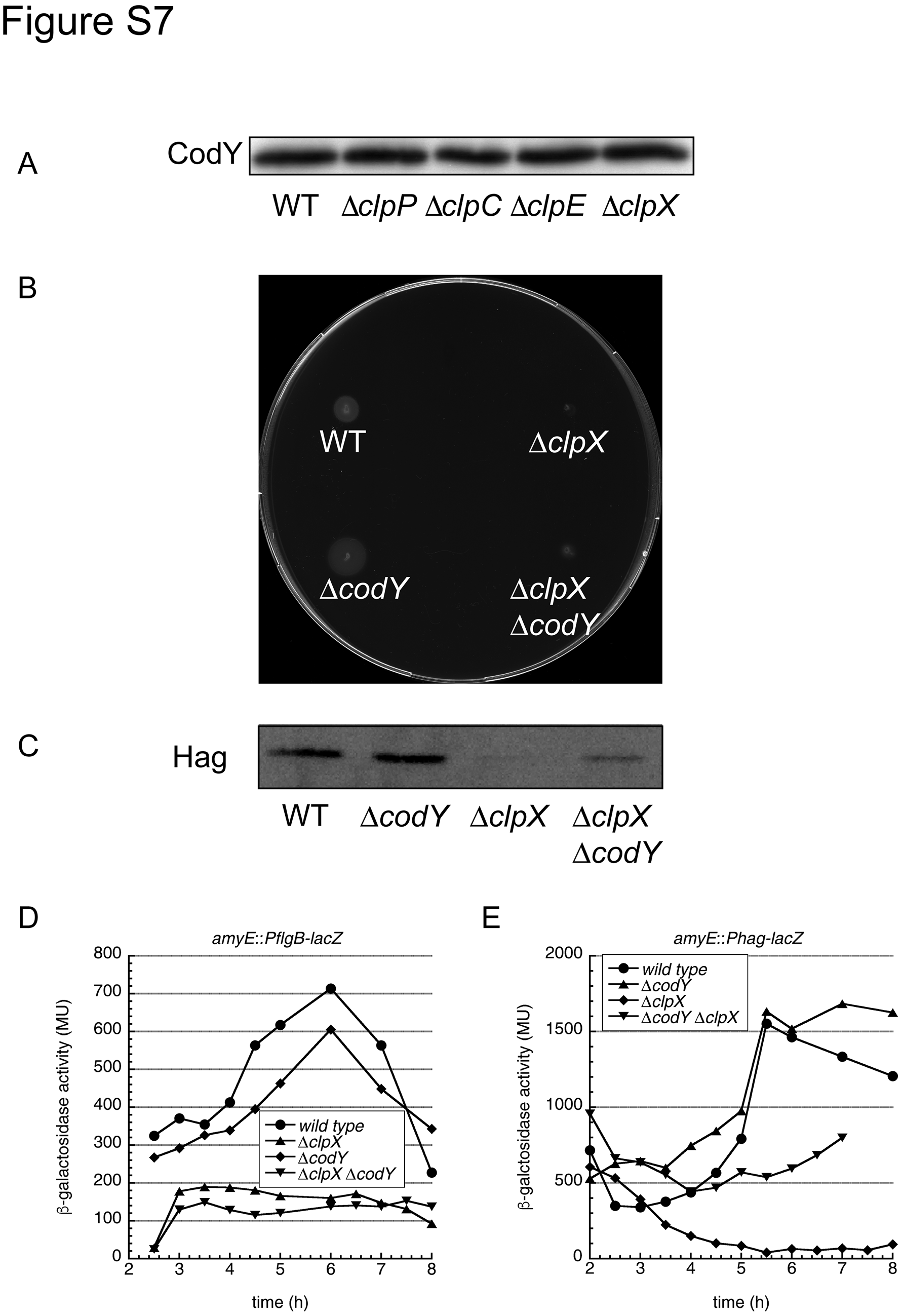

Supplement: Supplementary file 8 [file Image7.TIF]

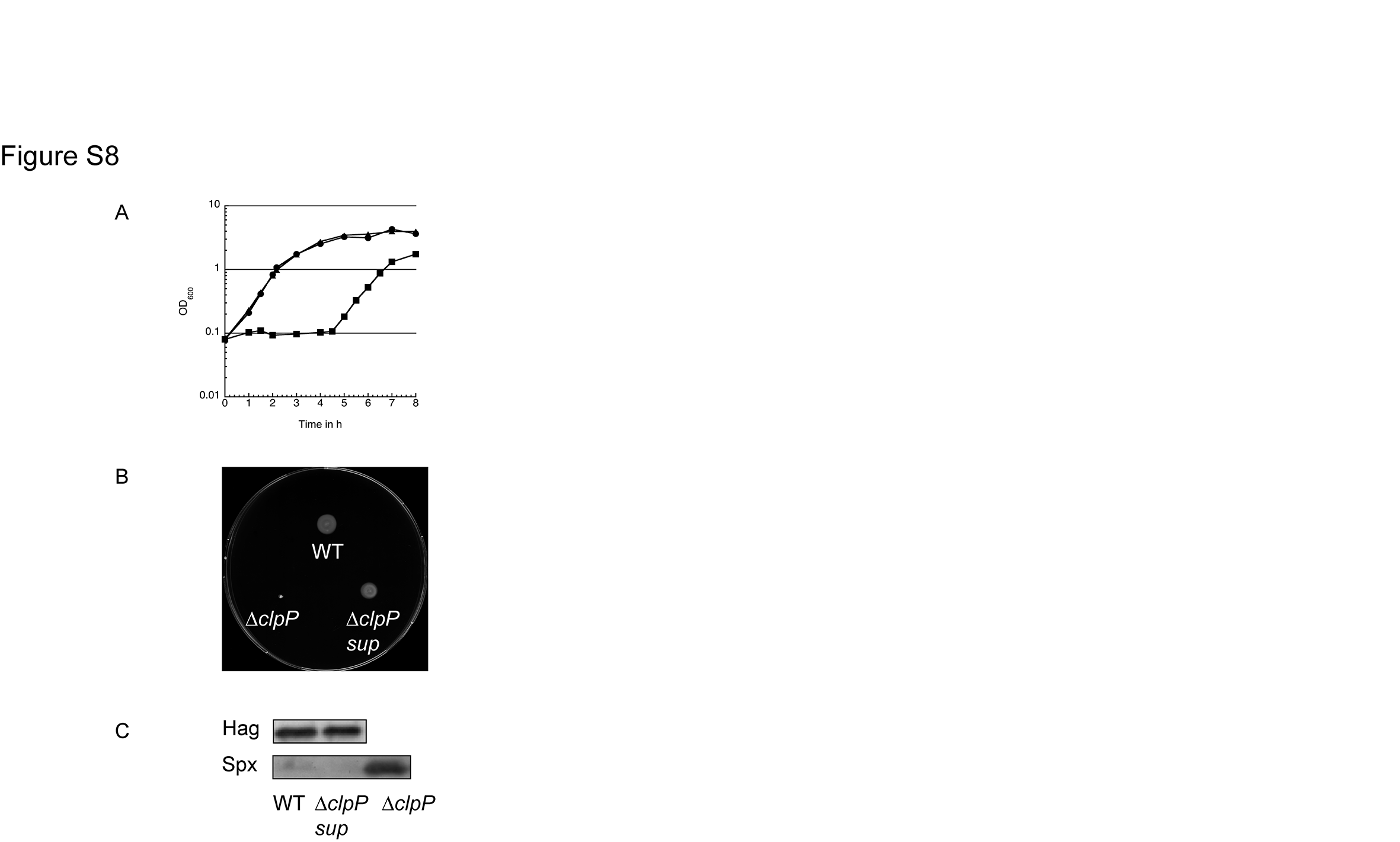

Supplement: Supplementary file 9 [file Image8.TIF]

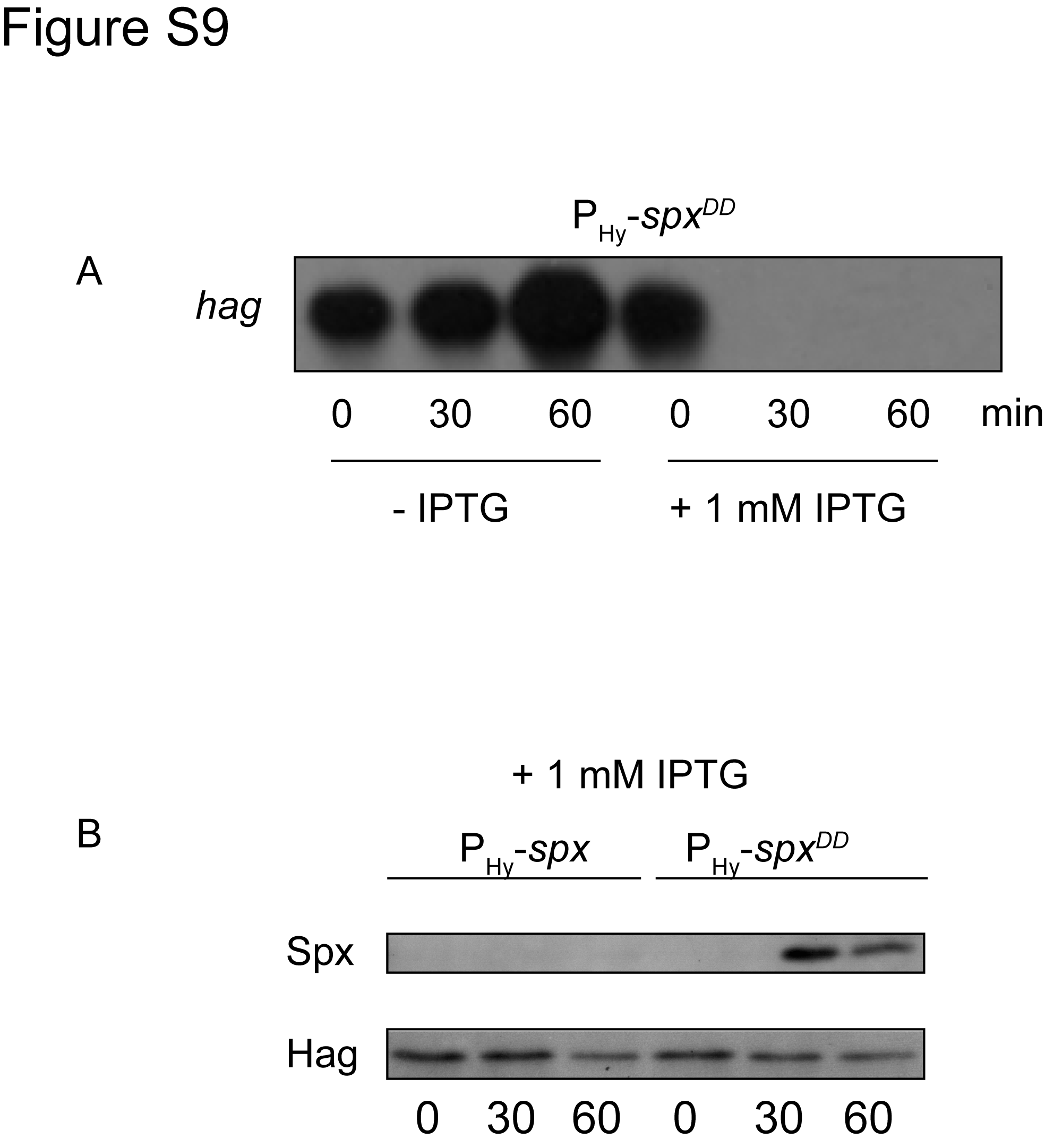

Supplement: Supplementary file 10 [file Image9.TIF]

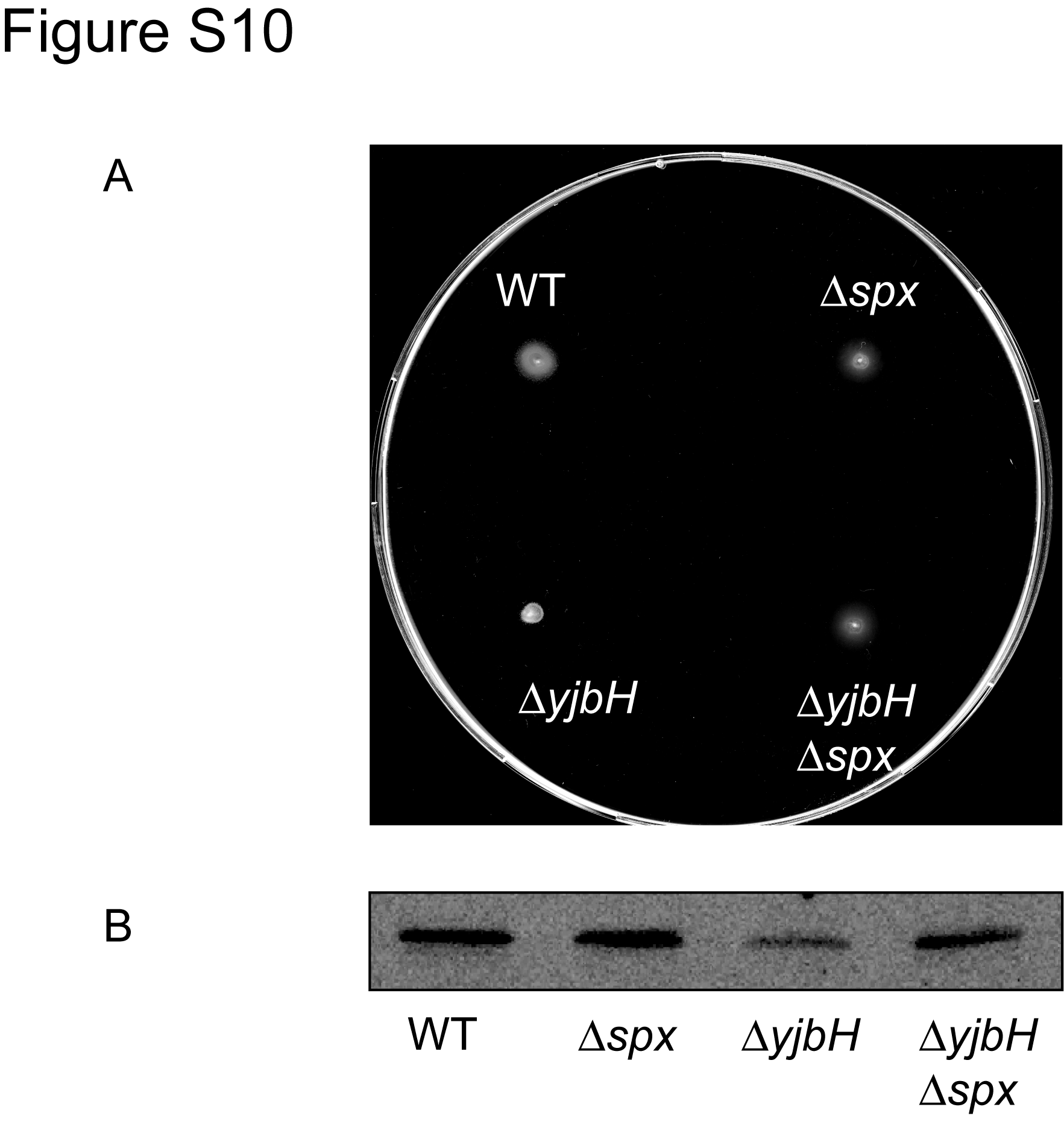

Supplement: Supplementary file 11 [file Image10.TIF]

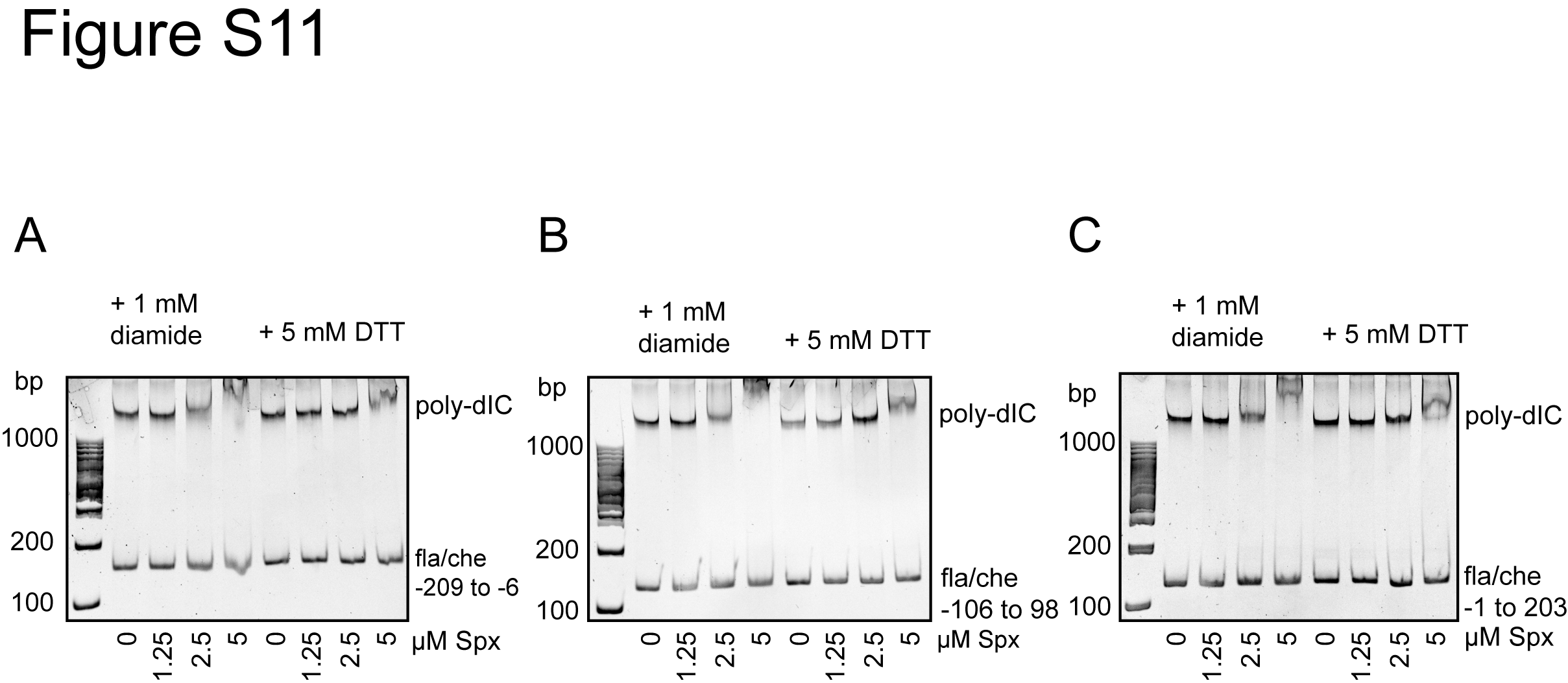

Supplement: Supplementary file 12 [file Image11.TIF]

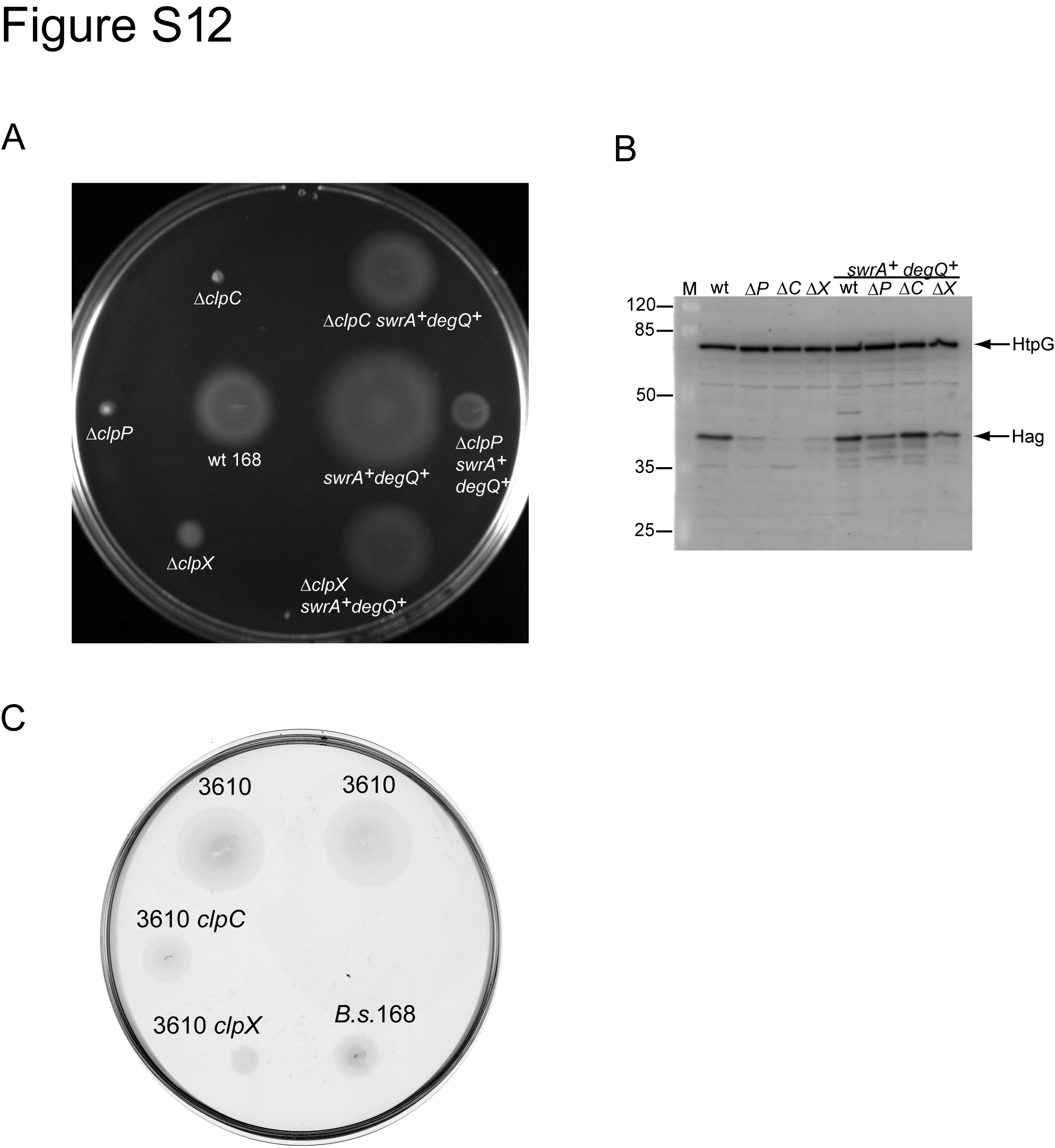

Supplement: Supplementary file 13 [file Image12.TIF]
